# Efficient perovskite solar modules enabled by a UV-stable and high-conductivity hole transport material

**DOI:** 10.1126/sciadv.adu3493

**Published:** 2025-05-28

**Authors:** Tianxiao Liu, Zhijun Ren, Yangyang Liu, Yaoyao Zhang, Jing Liang, Fangwen Cheng, Yiran Li, Xiaoyu Shi, Yunjie Dou, Xiaodong Hu, Lingyuan Wang, Siwei Luo, Feifei Wang, Xiaoxiao Peng, Yu Zhao, Wei Wang, Yi Cao, Feng Gao, Shangshang Chen

**Affiliations:** ^1^ State Key Laboratory of Coordination Chemistry, MOE Key Laboratory of High-Performance Polymer Materials & Technology, School of Chemistry and Chemical Engineering, Nanjing University, Nanjing, Jiangsu 21003, P. R. China.; ^2^ Department of Physics, Chemistry and Biology (IFM), Linköping University, Linköping SE-58183 Sweden.

## Abstract

Ultraviolet (UV) radiation poses a substantial challenge to the stability of prevalent p-i-n (positive-intrinsic-negative) perovskite solar cells (PSCs), demanding more robust hole-transport layers (HTLs) due to light incident from the HTL side. Here, we unveil that commonly used self-assembled monolayer (SAM)–type HTLs suffer from poor UV stability that causes irreversible damage to hole extraction and impairs device stability. To address this issue, we develop a polymeric and UV-stable HTL named Poly-2PACz, which exhibits strong binding to substrates and exceptional UV resistance over SAM-type HTLs. The PSCs blade-coated under ambient conditions using Poly-2PACz HTL achieved a remarkable efficiency of 26.0% and outstanding UV stability. Our cells retain 80% of the initial PCE even after about 500 hours of high-intensity UV illumination [7.7 times higher than that of air mass 1.5 global (AM 1.5G) solar spectrum]. Furthermore, Poly-2PACz exhibits good wettability and high conductance, enabling the fabrication of blade-coated minimodules with an aperture efficiency of 22.2% and excellent uniformity.

## Teaser

A polymeric and UV-stable hole-transport material enables high-performance air-processed perovskite solar cells and modules.

## INTRODUCTION

Metal halide perovskite solar cells (PSCs) have garnered extensive research interest due to their solution processibility, mechanical flexibility, and excellent optoelectronic properties (*1*–*3*). Among others, inverted PSCs with a positive-intrinsic-negative (p-i-n) structure are promising for commercial applications due to their simple fabrication and low cost (*4*–*7*). However, the commercialization of p-i-n PSCs is limited by their unsatisfactory long-term stability, particularly under real-world conditions where exposure to various environmental stress like heat (*8*), moisture (*9*), and ultraviolet (UV) radiation (*10*, *11*) can substantially impact device performance. Although the thermal and light (mostly visible light) stability of PSCs has been extensively investigated (*12*–*16*), the intrinsic UV stability remains less explored (*17*). UV light occupies only 4 to 5% of the air mass 1.5 global (AM 1.5G) solar spectrum, but it can induce deep-level defect states at interfaces (such as the transformation of Pb^2+^ to Pb^0^) that severely impair the operational stability of PSCs (*11*).

In single-junction p-i-n PSCs, solar light is incident from transparent conductive oxide (TCO)/hole-transport layer (HTL) side, making HTLs more susceptible to light degradation (*18*). Therefore, commercialization of inverted PSCs requires HTLs with excellent light stability, particularly UV stability. In addition, an ideal HTL should also efficiently transport holes and promote the formation of a uniform and well-crystallized perovskite film for scalable production (*19*–*21*). This is even more challenging when processing under ambient conditions, where environmental factors such as moisture, oxygen, and temperature can notably affect perovskite film morphology and microstructure (*22*, *23*).

Recent advances in self-assembled monolayers (SAMs), particularly carbazole phosphonic acids (PACz), have revolutionized hole transport in p-i-n PSCs, enabling them to surpass the efficiency of n-i-p architectures (*24*–*26*). SAM HTLs, composed of anchoring, spacer, and terminal groups, represent a breakthrough for p-i-n PSCs (*27*, *28*), with multiple cases of record-breaking PSCs achieved through their meticulous design and modification (*1*, *2*, *29*). Unlike conventional polymeric HTLs like PEDOT:PSS and PTAA, small-molecule PACz HTLs rely on SAM formation for hole extraction due to their inherently low conductivity. Their performance is sensitive to layer thickness and coverage, resulting in a narrow processing window and challenges in achieving uniformity over large areas. This makes a single SAM HTL difficult for mass production (*30*–*32*). Furthermore, perovskite devices require robust and light-stable HTLs due to light exposure (including UV light) from the HTL side. The UV stability of SAMs and their degradation mechanism remain rarely studied yet. A recent report from Wu and colleagues revealed that conjugated molecular structures show more effective electron/charge delocalization than nonconjugated MeO-2PACz (*14*). Their frontier energy level modulation strategy helps enhance SAM UV stability compared to nonconjugated PACz SAMs.

In this work, we investigate the UV stability of conventional PACz HTLs and identify the weak bonding between the phosphorous acid group and TCO together with the UV-caused decomposition as the primary factors leading to their poor irradiation stability. To overcome the limitations of PACz HTLs, we develop a polymeric HTL called Poly-2PACz by polymerizing PACz small molecules. In addition to strong UV stability compared with conventional PACz SAMs, Poly-2PACz also has high electrical conductivity and insensitivity to thickness variations, making it possible to develop highly efficient and stable large-area devices. Our PSCs (processed from the ambient blade-coating method) achieve an impressive efficiency of 26.0% (independently certified as 25.2%), setting a record for air-processed PSCs irrespective of fabrication methods or device structures. Notably, the minimodule efficiency based on Poly-2PACz reaches 22.2% with an aperture area of 12.25 cm^2^ (independently certified as 21.6%). Under a very harsh condition (continuous irradiation from a 365-nm UV lamp for approaching 500 hours), the efficiency of our PSCs remains at 80% of the initial value, greatly outperforming the control PSCs based on 2PACz. Even after 1500 hours of maximum power point (MPP) testing under AM 1.5G 1-sun full spectrum illumination, the initial efficiency is maintained at over 98%. Our polymeric HTL design strategy not only enables a major advance for the commercialization of PSCs but also opens up an avenue for HTL design toward more efficient and stable PSCs.

## RESULTS

**Fig. 1.**
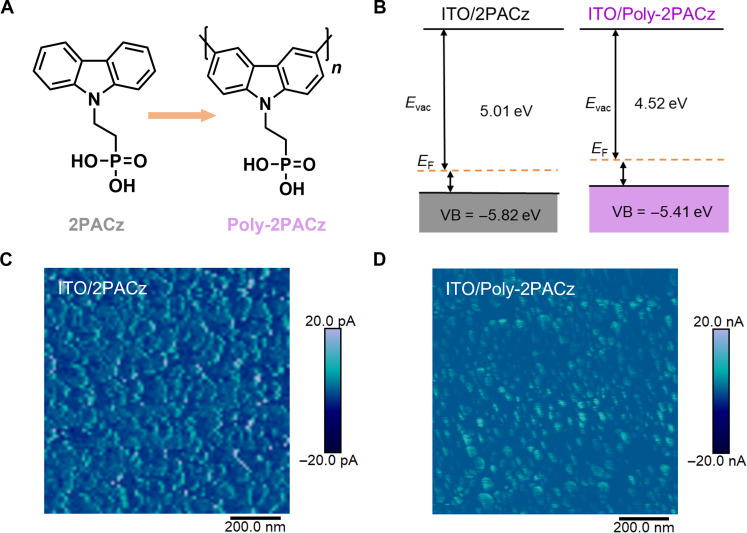
Characterizations of HTLs. (**A**) Chemical structures of 2PACz and Poly-2PACz. (**B**) Energy levels of 2PACz and Poly-2PACz thin films on ITO measured by UPS. (**C** and **D**) c-AFM current images of ITO glass substrates covered by 2PACz (C) and Poly-2PACz (D).

The chemical structures of 2PACz and Poly-2PACz are shown in Fig. 1A, and the synthesis details of Poly-2PACz can be found in the Supplementary Materials. In brief, brominated carbazole phosphonate was first obtained by the reaction between 3,6-dibromocarbazole and diethyl(2-bromoethyl)phosphonate, followed by Yamamoto polymerization reaction to yield poly(carbazole phosphonate). Last, Poly-2PACz was obtained through the hydrolysis of phosphonate to phosphorus acid. Notably, all starting materials used in the synthesis are inexpensive, and the reactions were conducted under mild conditions. Poly-2PACz thin film displays a bathochromically shifted absorption spectrum compared to 2PACz (fig. S1). This shift is caused by the extended conjugation formed along the polymer backbone after polymerization. To investigate the interaction between Poly-2PACz and the indium tin oxide (ITO) glass substrate, x-ray photoelectron spectroscopy (XPS) analysis was conducted on both bare ITO and ITO/Poly-2PACz substrates (*33*). A noticeable shift in In 3d peaks (fig. S2) was detected after depositing Poly-2PACz onto the ITO surface, providing evidence for the successful binding of Poly-2PACz to ITO through the phosphoric acid groups.

To investigate the effects of the polymerization strategy on HTL energy levels, UV photoelectron spectroscopy (UPS) characterization was used to determine the valence band (VB) of the HTLs on ITO. The VB levels of ITO/2PACz and ITO/Poly-2PACz were measured to be −5.82 and −5.41 eV, respectively (Fig. 1B and fig. S3), indicating that the polymerization strategy upshifts the VB level. Notably, the VB level of Poly-2PACz aligns better with those (−5.4 eV) of MAPbI_3_ or FAPbI_3_ perovskite compositions. Atomic force microscopy (AFM) was then used to analyze their surface topography. As shown in fig. S4, AFM height images reveal a smooth surface for the ITO/Poly-2PACz substrate, with a root mean square (RMS) roughness of 2.9 nm, similar to that of the ITO/2PACz substrate (3.1-nm RMS roughness). The Poly-2PACz–coated ITO substrate shows a smaller contact angle than 2PACz (fig. S5), which is beneficial for the spreading of perovskite inks during scalable fabrication (*29*).

Conductivity is a critical figure of merit for an HTL. To compare their conductance, conductive AFM (c-AFM) characterization was performed and shows a marked difference in their conductivity (Fig. 1, C and D). Under a low bias voltage of only 5 mV, Poly-2PACz exhibits an average current (2.4 nA) that is three orders of magnitude higher than 2PACz (2.2 pA, measured at 5 V). This finding confirms the superior conductivity of Poly-2PACz compared to conventional PACz-type SAMs. The inherent high conductivity of Poly-2PACz enables efficient hole transportation even in thick layers, representing a notable advancement in large-scale fabrication compared to conventional 2PACz-type SAMs.

**Fig. 2.**
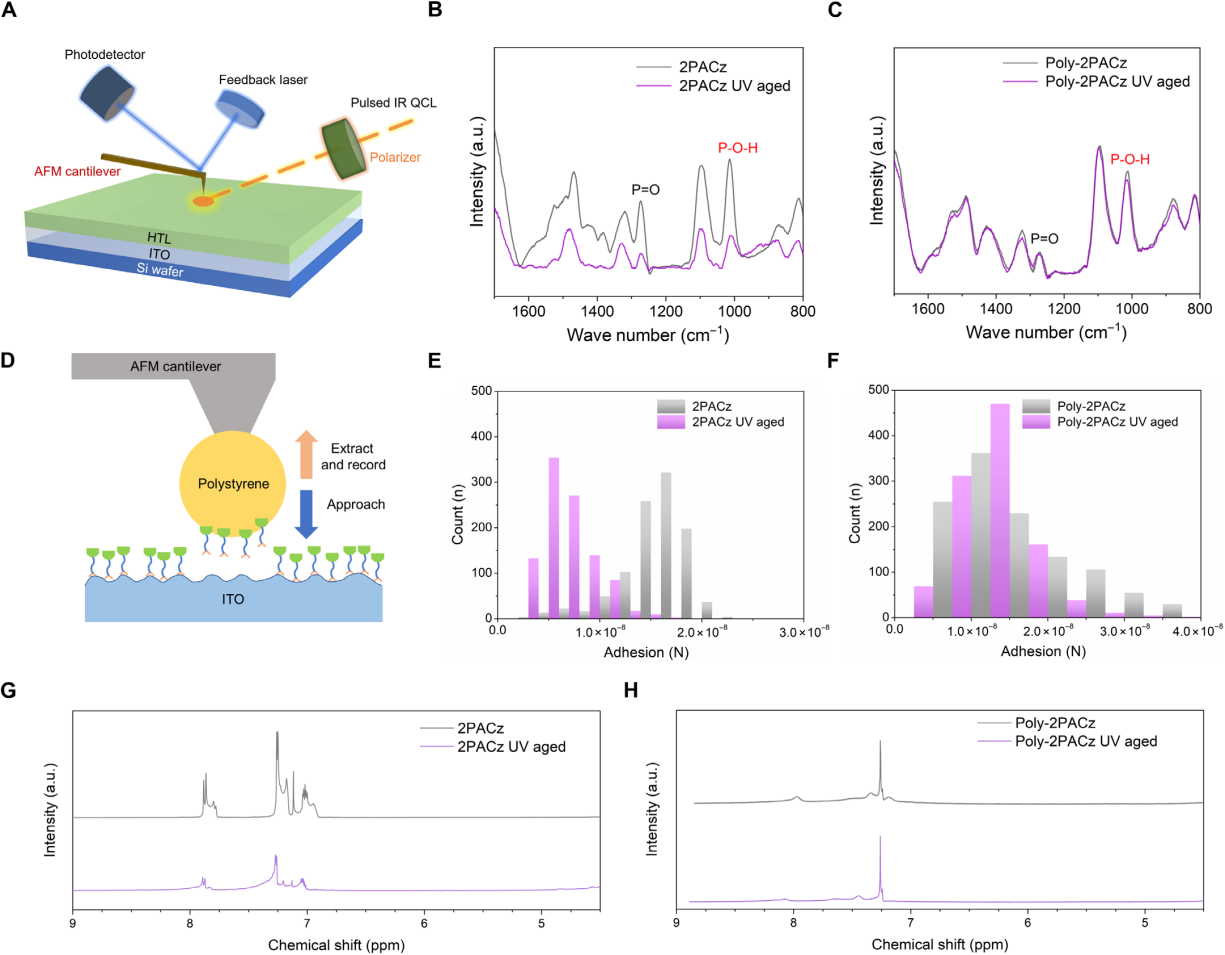
UV stability of HTLs. (**A**) Schematic illustration of IR-PiFM measurement on Si/ITO wafer coated with HTLs. (**B** and **C**) IR-PiFM spectra of 2PACz (B) and Poly-2PACz (C) on Si/ITO substrates before and after 24-hour UV irradiation (365 nm, 17.0 mW cm^−2^). a.u., arbitrary units. (**D**) Schematic illustration of AFM adhesion-force measurement on ITO glass substrates coated with HTLs. (**E** and **F**) Adhesion force distributions of 2PACz (E) and Poly-2PACz (F) on ITO glass substrates before and after 24-hour UV irradiation (365 nm, 17.0 mW cm^−2^) determined from AFM adhesion-force measurement. (**G** and **H**) ^1^H NMR spectra of 2PACz (G) and Poly-2PACz (H) before and after 48-hour UV irradiation.

We further investigate the UV stability of these two HTLs on ITO glass substrates using infrared photoinduced force microscopy (IR-PiFM; Fig. 2A), allowing us to probe interface binding changes before and after UV light exposure. 2PACz- and Poly-2PACz–coated ITO glass substrates were soaked under a 365-nm UV lamp with an intensity of 17.0 mW cm^−2^. Their pristine and aged IR-PiFM spectra are shown in Fig. 2 (B and C). For the 2PACz thin layer, the IR peaks at 1015 and 1275 cm^−1^, corresponding to vibration peaks of P─O─H and P═O groups, exhibited a decrease in absorbance after 24 hours of high-dose UV soaking (Fig. 2B). This suggests a substantial weakening of the bonds between 2PACz and ITO, leading to the desorption of 2PACz molecules from the ITO substrate under UV irradiation. In addition to 2PACz, we also characterized other benchmark SAM HTLs including Me-4PACz, MeO-2PACz, and Ph-4PACz (*34*). All these SAM HTLs exhibited a similar trend after UV soaking (fig. S6). It appears that most reported carbazole-based SAM HTLs suffer from degraded bonding to substrates under UV stimulation, thus limiting the light stability of the PSCs based on these SAM HTLs. In notable contrast, Poly-2PACz exhibited excellent UV stability, with no notable changes observed in IR absorbance (Fig. 2C). The high molecular weight of Poly-2PACz polymer chains likely enables it to adhere more stably to ITO substrates, making them less prone to migration or detachment upon UV exposure.

To investigate the adhesion force between 2PACz and Poly-2PACz with the underlying ITO substrate, AFM adhesion-force measurements were used using a polystyrene (PS)–modified cantilever (Fig. 2D; details in the Characterizations section) (*35*–*37*). Before UV exposure, Poly-2PACz exhibited a slightly stronger average adhesion force (1.76 × 10^−8^ N) compared to 2PACz (1.58 × 10^−8^ N), indicating a more robust bonding interaction with the ITO. The improved adhesion of Poly-2PACz results from a synergistic interaction between its functional binding motifs along the polymer backbone and the presence of sites that remain attached. These attached sites effectively limit the diffusion of detached motifs, facilitating rebinding and enhancing the overall adhesive strength (*38*–*40*). This process enables greater energy dissipation prior to rupture, thereby contributing to the superior adhesive performance of Poly-2PACz. Notably, after 24 hours of UV irradiation, an obvious difference emerged: The adhesion force of the 2PACz HTL nearly halved, whereas Poly-2PACz displayed minimal degradation (Fig. 2F).

To understand the degradation mechanism of 2PACz-type SAMs under UV irradiation, we first conducted proton nuclear magnetic resonance (^1^H NMR) tests on both 2PACz and Poly-2PACz before and after 48 hours of UV irradiation, as shown in Fig. 2 (G and H). 2PACz exhibited notable changes in the aromatic region. The decrease in intensity of the characteristic aromatic ring signals between 7 and 8 parts per million (ppm) indicates the cleavage of the carbazole ring, signifying the photoinstability of 2PACz. In contrast, Poly-2PACz showed negligible changes in its spectrum after UV soaking, confirming the preservation of its overall molecular structure. To further investigate the impact of UV irradiation on the bonding between the HTL and ITO, we performed XPS characterization on the HTL-coated ITO before and after UV irradiation. As shown in fig. S7, after UV irradiation, the peak of the P element in the 2PACz film shifted toward a lower binding energy, and its intensity notably decreased. In contrast, the intensity of the P element in the Poly-2PACz film remained unchanged, consistent with the IR-PiFM results. Overall, UV irradiation has two detrimental effects on the stability of small-molecule SAMs. First, high-energy UV irradiation causes the decomposition of the aromatic rings of the SAMs. Second, UV light weakens the bonding between the phosphonic acid groups and ITO, leading to their desorption from the ITO surface. In contrast, Poly-2PACz, a polymeric HTM, exhibits enhanced UV stability due to the delocalization of electron density along its conjugated polymer backbone. In addition, the stronger adsorption of Poly-2PACz to ITO minimizes desorption under UV soaking.

**Fig. 3.**
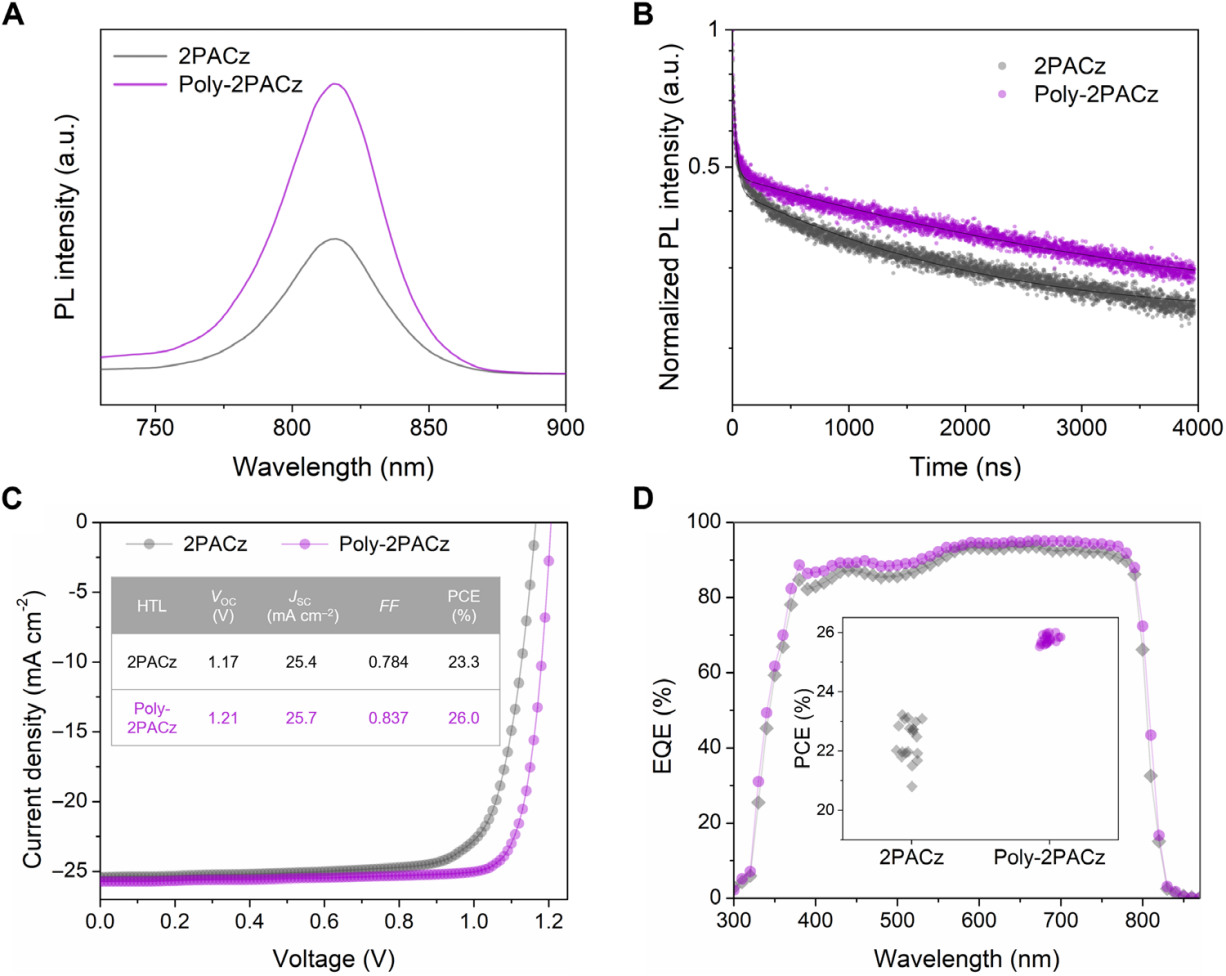
Effects of HTLs on film PL properties and device PCEs. (**A** and **B**) Steady-state PL (A) and TRPL spectra (B) of perovskite films coated on 2PACz and Poly-2PACz. (**C**) Backward *J*-*V* characteristic curves of the champion PSCs based on 2PACz and Poly-2PACz. Inset shows the photovoltaic parameters of the champion PSCs. (**D**) EQE spectra of the champion PSCs based on 2PACz and Poly-2PACz. Inset shows the PCE distribution of 2PACz and Poly-2PACz–based PSCs.

Perovskite films were blade coated onto the top of HTL-coated ITO substrates, and the effects of HTLs on perovskite quality were then investigated. The x-ray diffraction (XRD) patterns show a stronger peak at 13.9° for the perovskite film on Poly-2PACz than on 2PACz (fig. S8). The surface morphology of the perovskite films examined with AFM characterization (fig. S9) shows notable grain enlargement and improved uniformity of Poly-2PACz–based perovskite film. These suggest that the better wettability of Poly-2PACz also facilitates the uniform nucleation and crystallization of perovskite films. Further photoluminescence (PL) (Fig. 3A) and time-resolved photoluminescence (TRPL) (Fig. 3B) characterizations show that the perovskite film coated on Poly-2PACz exhibited enhanced PL intensity by two times and prolonged PL lifetime (2907 ns versus 1552 ns; table S1), confirming the better passivation effects of Poly-2PACz in suppressing the nonradiative recombination centers (*41*, *42*).

Subsequently, p-i-n structured perovskite devices were fabricated with a planar architecture of glass/ITO/HTL/MA_0.7_FA_0.3_PbI_3_/C_60_/bathocuproine (BCP)/copper (Cu). The current density-voltage (*J*-*V*) characteristic curves of PSCs are shown in Fig. 3C. The blade-coated PSCs based on 2PACz achieved a decent power conversion efficiency (PCE) of 23.3%, along with an open-circuit voltage (*V*_OC_) of 1.17 V, a short-circuit current density (*J*_SC_) of 25.4 mA cm^−2^, and a fill factor (*FF*) of 0.784. In contrast, the champion efficiency reached 26.0% for the Poly-2PACz–based device, with a *V*_OC_ of 1.21 V, a *J*_SC_ of 25.7 mA cm^−2^, and an *FF* of 0.837. To the best of our knowledge, this sets the record PCE of air-processed PSCs irrespective of fabrication methods or device structures. Our best-performing PSC based on Poly-2PACz was sent to an accredited certification center (Shanghai Institute of Microsystem and Information Technology), where a comparable efficiency of 25.2% (derived from backward *J*-*V* curve) and a stabilized PCE of 25.0% [300-s MPP tracking (MPPT)] were confirmed (fig. S10). Compared with 2PACz, all photovoltaic parameters of Poly-2PACz–based cells are substantially enhanced. The elevated *V*_OC_ can be partially attributed to the reduced nonradiative recombination as confirmed by the PL and TRPL results, and the high conductivity contributed to the improved *FF*. The integrated current density of the Poly-2PACz–based device (25.2 mA cm^−2^) in the external quantum efficiency (EQE) characterization (Fig. 3D) is also higher than that of the 2PACz-based device (24.8 mA cm^−2^), indicating the overall high photo-to-current conversion efficiency from 400 to 800 nm. In addition, the PSC based on Poly-2PACz exhibited reduced hysteresis compared to that of 2PACz (figs. S10 and S11). Table S2 and fig. S12 show the statistical analysis of device parameters, and the average PCEs of 2PACz- and Poly-2PACz–based PSCs are analyzed to be (22.6 ± 0.7) and (25.9 ± 0.1)%, respectively, confirming the good reproducibility of Poly-2PACz–based cells.

Moreover, the 2PACz HTL relies on forming thin SAM layers and exhibits high sensitivity to layer thickness. When the 2PACz solution was blade coated at different concentrations, we found that even small changes in concentration resulted in a notable fluctuation in overall photovoltaic parameters, with decent PCEs only obtained at a low concentration of 0.5 mg ml^−1^ (fig. S13). On the contrary, there is minimal variation in device parameters when processing Poly-2PACz with concentrations ranging from 0.3 to 3 mg ml^−1^. Its insensitivity to thickness and wide processing window make it highly desirable for the fabrication of large-area modules (*18*).

**Fig. 4.**
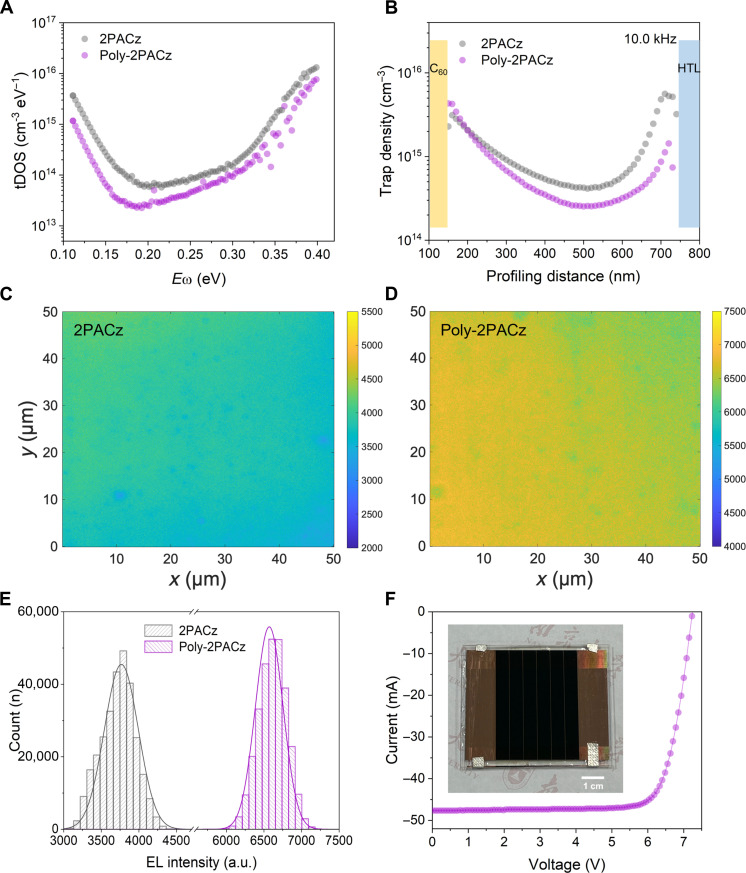
Device characterizations. (**A** to **E**) tDOS spectra (A), DLCP trap profiling (10.0 kHz, B), EL mapping [(C) and (D)], and EL intensity distributions (E) of the PSCs based on 2PACz and Poly-2PACz. (**F**) *I*-*V* curve of champion perovskite minimodule (12.25-cm^2^ aperture area) based on Poly-2PACz. Inset shows a photograph of the champion perovskite minimodule based on Poly-2PACz.

A battery of device characterizations was then performed to elucidate the performance enhancement mechanism of Poly-2PACz–based devices. Thermal admittance spectroscopy was used to characterize the cell trap density with different energy depths (*43*–*45*). As shown in trap density of state (tDOS) spectra (Fig. 4A), the Poly-2PACz–based device exhibited a lower overall trap density than the 2PACz counterpart. To further investigate how Poly-2PACz reduced trap density spatially, drive-level capacitance profiling (DLCP) measurement was used to map the spatial distribution of trap states inside the PSCs in Fig. 4B. An obvious reduction in defects at the HTL/perovskite interface was observed after replacing 2PACz with Poly-2PACz at 10 kHz (deep trap responding), indicating the effectiveness of Poly-2PACz in passivating the interface deep traps, which was studied to have critical effects on cell PCEs and stability (*46*). We further carried out electroluminescence (EL) mapping of the complete PSCs to investigate the microhomogeneity of blade-coated perovskite films on 2PACz and Poly-2PACz. Different from conventional PL mapping that only provides insight into the photoactive layers, EL mapping allows the characterization of a solar cell in a state close to its operational condition (*47*, *48*). In addition, PL mapping typically suffers from interference from lateral carrier diffusion in the proximity of the excitation spot. Complementing PL mapping, EL mapping is able to quantitatively measure the luminescence flux of completed cells and was found to be free from a variety of artifacts occurring from the analysis in a confocal configuration (*49*, *50*). A forward bias was applied to stimulate luminescence. The size of each EL detection zone is 50 μm by 50 μm, and the resolution of each pixel is 160 nm. As shown in EL maps (Fig. 4, C and D), the PSC based on Poly-2PACz exhibited both higher EL intensity and better EL emission homogeneity (Fig. 4E), whereas 2PACz-based cell suffered from much poorer EL emission characterized by a few dispersed dark centers. These results confirm the advantages of Poly-2PACz over conventional 2PACz-type SAMs in terms of uniform deposition.

Leveraging the excellent performance of Poly-2PACz in small-area devices, we further used blade-coating to fabricate large-area modules under ambient conditions. As expected from the high uniformity of Poly-2PACz films on ITO, the individual cells within the module exhibited minimal performance variation. We achieved a remarkable aperture-area efficiency of 22.2% on a 12.25-cm^2^ module, with a high *V*_OC_ of 7.24 V (six subcells in series), a short-circuit current (*I*_SC_) of 47.8 mA, and an *FF* of 0.787 (Fig. 4F). Independent certification from an accredited measurement center confirmed a comparable aperture PCE of 21.6% (fig. S14). Notably, the geometric filling factor (GFF) of our perovskite module was measured to be 94.4% (fig. S15), and thus the active-area PCE of our module reached 23.5% correspondingly. Furthermore, the calculated *V*_OC_ of each subcell (1.21 V) is identical to that of the small-area PSCs. This minimal efficiency difference between cells and modules, coupled with the maintained *V*_OC_, underscores the exceptional uniformity and scalability of Poly-2PACz for large-scale perovskite module fabrication.

**Fig. 5.**
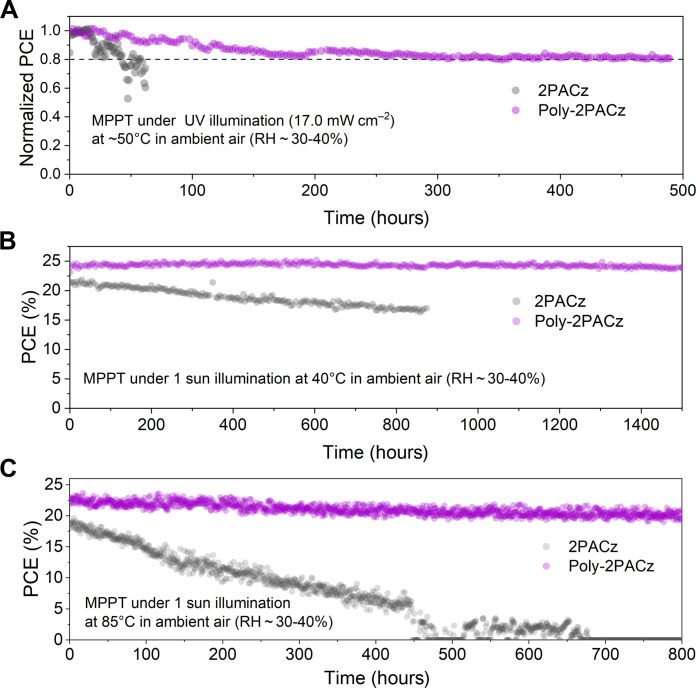
Stability study. (**A** to **C**) MPP stability test results of the encapsulated PSCs soaked under a 365-nm UV lamp (17.0 mW cm^−2^) at ~50°C (A), 1-sun illumination (100 mW cm^−2^) at 40°C (B), and 1-sun illumination (100 mW cm^−2^) at 85°C (C). RH, relative humidity.

To evaluate the light stability, a critical photovoltaic parameter for a solar cell, we first assessed the long-term UV tolerance of the encapsulated PSCs using an automatic MPP testing system. Under continuous UV irradiation (365 nm, 17.0 mW cm^−2^) at ambient conditions, the Poly-2PACz device displayed remarkably enhanced UV stability. It retained an impressive 80% of its initial efficiency after 480 hours of irradiation (Fig. 5A), which translates to a UV dose of 81.6 kilowatt-hour (kWh) m^−2^ and is 5.4 times greater than that of IEC61215 standard (*51*). We further investigated their operational stability under simulated sunlight (AM 1.5G 1-sun illuminance, 100 mW cm^−2^) at 40°C. Impressively, the Poly-2PACz cell maintained 98% of its initial efficiency after 1500 hours of continuous light exposure, whereas the 2PACz-based devices exhibited a continuous decline (Fig. 5B). We also tested their operational stability at an elevated temperature of 85°C, and Poly-2PACz–based cell was still able to maintain 89% of PCE after 800-hour MPPT (Fig. 5C), remarkably outperforming that based on 2PACz. These findings strongly support the effectiveness of Poly-2PACz in improving the stability of PSCs under both UV stimulation and solar light soaking conditions.

## DISCUSSION

In conclusion, we successfully developed a strategy to address the UV stability challenge of state-of-the-art SAMs. A conductive polymer, Poly-2PACz, was developed with high electrical conductivity, good wettability, and excellent UV stability. Poly-2PACz effectively improves perovskite film uniformity and suppresses recombination, as evidenced by EL mapping and trap-related characterizations. Consequently, the resulting PSCs achieved an impressive efficiency of 26.0% and retained 80% of their initial efficiency after high-dose UV irradiation for 480 hours. Furthermore, the hydrophilic and uniform Poly-2PACz surface facilitates scalable perovskite film coating, enabling the production of minimodules with a high aperture PCE of 22.2%. Overall, this work presents an approach to perovskite HTL design and holds promise for the practical manufacturing of perovskite solar modules.

## MATERIALS AND METHODS

### Materials

BCP, lead iodide (PbI_2_, 99.999% trace metals), dimethyl sulfoxide (DMSO), l-α-phosphatidylcholine (LP), benzylhydrazine hydrochloride (BHC), 2-methoxyethanol (2-ME), and toluene were purchased from Merck and used without further purification. C_60_ was purchased from Shenzhen eFlex Inc. Methylammonium iodide (MAI), formamidinium iodide (FAI), 4-fluoro-phenethylammonium iodide (p-F-PEAI), and *n*-dodecylammonium iodide were purchased from Great Cell Solar. 2PACz, Me-4PACz, MeO-4PACz, and Ph-4PACz were purchased from Derthon Optoelectronics Materials Science Technology Co Ltd.

### Device fabrication

Patterned ITO glass substrates (1.5 cm by 1.5 cm) were first cleaned by ultrasonication with soap, deionized water, and isopropyl alcohol and then UV-ozone treated for 15 min before use. All PSCs were prepared by blade-coating at room temperature inside a fume hood with a relative humidity of 30 to 40%. The HTL solution (2PACz in methanol or Poly-2PACz in methanol:chloroform = 1:1) was blade coated onto ITO glass substrates at a speed of 20 mm s^−1^, followed by the thermal annealing at 100°C for 10 min in air. The gap between the blade-coater and ITO substrates was 150 μm. A 1.35 M MA_0.7_FA_0.3_PbI_3_ precursor solution was prepared by dissolving corresponding organic halide salts and lead iodide into 2-ME in an N_2_-filled glovebox with an O_2_ level of <10 ppm. A solution of *n*-dodecylammonium iodide (0.83 mg ml^−1^), LP (0.27 mg ml^−1^), 0.14% (v/v) MAH_2_PO_2_, p-F-PEAI (1.4 mg ml^−1^), BHC (0.15 mg ml^−1^), and 2.8% (v/v) DMSO was added as additives before blade-coating. Subsequently, the precursor solution was blade coated onto the HTL-covered ITO glass substrates with a gap of 230 μm at a movement speed of 20 mm s^−1^. The air knife worked at 20 psi (138 kPa) during blade-coating. After that, the perovskite films were annealed at 120°C for 10 min in air to remove the residual solvents. The solar cells were completed by thermally evaporating C_60_ (30 nm 0.3 Å s^−1^), BCP (6 nm, 0.1 Å s^−1^), and copper (100 nm, 1 Å s^−1^). The PSCs were then encapsulated with cover glass sealed by a two-part epoxy encapsulant. The active area of solar cells is 8.0 mm^2^. For perovskite minimodules, the laser scribing was performed twice with a UV laser marker (355 nm). The laser power for P2 and P3 scribing is ~0.375 W. The champion module based on Poly-2PACz has six subcells, and each subcell has a width of 6 mm. The total scribing linewidth was ~0.336 mm, giving a GFF of 94.4%. A polydimethylsiloxane layer prepared by a soft lithography method was applied to the front surface of glass substrate as antireflection coating during the measurement of the perovskite solar modules.

### Characterizations

UV-vis absorption spectra were obtained with a Shanghai Metash UV-8000 spectrometer. UPS spectra were acquired with a UV photoelectron spectrometer (Surface analysis Kratos Axis Ultra DLD) under a vacuum of 2 × 10^−8^ mbar. The incident photon energy (He I) was 21.22 eV, and a −10-V bias was applied on the samples to enhance the signals of with kinetic energy near zero. XPS was performed using a Thermo SCIENTIFIC ESCALAB Xi+ with Al Kα anode source (1486.6 eV) operated at 15 kV and 10^−9^ mbar chamber pressure. AFM and c-AFM images were obtained with a Bruker Icon atomic force microscope. IR-PiFM spectra were recorded on a VistaScope system (Molecular Vista, USA). The VistaScope microscope is coupled to a quantum cascade laser (QCL) system from Block Engineering with a wave number resolution of 1 cm^−1^ and a tuning range from 770 to 1885 cm^−1^. The p-polarized pulsed QCL was focused at the tip end of a Au-coated cantilever through an integrated off-axis parabolic mirror. The set point was set as 85% with an oscillation amplitude of ∼1 nm. The cantilever was excited at its second resonance around 1.6 MHz. The time per image was about 4.5 min at 256 × 256 resolution. The spectrum was normalized with the background laser power profile spectrum. Multifrequency tapping mode AFM operation was implemented. The PiFM heterodyne mode was modulated to detect the surface, and the modulated repetition frequency of QCL is defined as the difference (*f*_m_ = *f*_2_ − *f*_1_) between the first (*f*_1_) and second (*f*_2_) eigenmodes of the cantilever; *f*_1_ was used to detect the PiFM signal, whereas *f*_2_ was used to detect the topography. PiFM homodyne mode was modulated to detect the volume, which defined *f*_m_ as *f*_m_ = *f*_1_. For the bias-controlled experiments, the scanning direction was consistent with the electric field direction. As for AFM adhesion-force measurement, excess solvents were used to rinse the surface of HTL-coated ITO glass substrates to make sure the hole transporters formed a single layer. After that, the HTL-coated ITO glass substrate was glued onto a glass slide, and an SNL-10 cantilever (Type A, from Bruker, Camarillo, CA, USA) was used for the adhesion-force measurements. A PS sphere (20 μm in diameter) was attached to the cantilever using epoxy. The adhesion-force measurements were conducted on a commercial AFM (NanoWizard IV from JPK, Germany). We randomly selected three areas on sample surface for AFM adhesion-force measurements, and in each area, a 20 × 20 data point matrix was set up, with force measurements performed at each data point. During a typical AFM measurement, the PS sphere-modified cantilever was approached to the sample surface at a constant speed of 2 μm s^−1^ until the target set point of 0.5 nN was reached. The cantilever was held at 0.5 nN on the sample surface for 2 s before being retracted at the same speed. The force-extension curves were recorded and analyzed using commercial software (JPK Data Processing, from JPK, Germany). Both steady-state PL and TRPL spectra were acquired on a HORIBA FL-3 fluorescence spectrophotometer at room temperature. The excitation wavelength was 405 nm for both PL and TRPL measurements. The imaging system of EL mapping was conducted using an inverted microscope (Eclipse Ti-U, Nikon) with an Autolab potentiostat (PGSTAT302N). A forward voltage was applied to stimulate luminescence. The EL light from the cell was captured by a charge-coupled device camera (Stingray, Allied Vision Technologies) through a 40X objective (numerical aperture = 0.6). A data acquisition card (USB-6281, National Instruments) was used to synchronize the voltage output from the potentiostat and transistor-transistor logic signals from the camera. The *J*-*V* characteristics of solar devices were performed using an LED 3A solar simulator (BG-LED3A-100S, Class AAA Solar Simulator) in air, and the power of the simulated light was calibrated to 100 mW cm^−2^ by a silicon reference cell (Newport 91150 V). All devices were measured using a Keithley 2400 source meter with a backward scan rate of 0.1 V s^−1^ from 1.25 to −0.2 V in air at room temperature, and the delay time was 10 ms. The aperture area is 7.5 mm^2^. There was no preconditioning before measurement. The Zolix DSR-600 EQE system was used to measure EQE spectra, which was calibrated by a standard Si diode. Monochromatic light was generated from a GLORIA-X150A xenon lamp. tDOS spectra and DLCP profiles were obtained with an Agilent E4980A precision LC meter. Photostability of the devices was performed using an automatic MPP tracker (91 PVKSOLAR). The encapsulated devices were measured under continuous illumination by a solar simulator (100 mW cm^−2^) under air conditions (the relative humidity was ~30 to 40%). The UV stability testing was conducted using a 365-nm UV lamp with an intensity of 17.0 mW cm^−2^ measured by a PM100A-Thorlabs photodetector. The encapsulated devices were connected to the automatic MPP tracker and soaked under continuous UV illumination at the same ambient conditions. In terms of UV intensity analysis, the AM 1.5G 1-sun standard illuminance (100 mW cm^−2^) contains a UV intensity (<365 nm) of 4.6 mW cm^−2^. Our UV light intensity (17.0 mW cm^−2^) is over three times higher than that in AM 1.5G 1-sun spectrum. Our UV soaking test involved a total UV dose of 81.6 kWh/m^2^ for about 500 hours. To estimate the real-world equivalent of this dose, we consulted the Global Solar Atlas (https://globalsolaratlas.info/map) for annual solar irradiance data. Solar irradiance varies by region due to factors like geography and climate. We focused on the 30° to 40° latitude band in China, which is close to the 37° latitude used in the AM 1.5G spectrum. In this region, the average annual global horizontal irradiance is around 1350 kWh/m^2^ (e.g., 1306 kWh/m^2^ in Nanjing and 1401 kWh/m^2^ in Beijing). Given that UV light typically represents ~4.6% of total solar irradiance, the annual UV dose in this region is roughly 62 kWh/m^2^. Therefore, our accelerated UV soaking test simulates the amount of UV exposure a device would receive in ~1.3 years under real-world conditions.

## Supplementary Materials

### This PDF file includes:

Synthesis of Poly-2PACz Figs. S1 to S19 Tables S1 and S2
