# Supplementary material for: Efficient perovskite solar modules enabled by a UV-stable and high-conductivity hole transport material
Source: Sci Adv. 2025 May 28;11(22):eadu3493. doi: 10.1126/sciadv.adu3493 (PMC12118634; doi:10.1126/sciadv.adu3493)
Supplement: Supplementary file 1 — Synthesis of Poly-2PACz Figs. S1 to S19 Tables S1 and S2 [file sciadv.adu3493_sm.pdf]

Science Advances
AAAS

Supplementary Materials for

Efficient perovskite solar modules enabled by a UV-stable and high-conductivity hole transport material

Tianxiao Liu *et al.*

Corresponding author: Feng Gao, feng.gao@liu.se; Shangshang Chen, schen@nju.edu.cn

Sci. Adv. **11**, eadu3493 (2025)
DOI: 10.1126/sciadv.adu3493

This PDF file includes:

Synthesis of Poly-2PACz
Figs. S1 to S19
Tables S1 and S2

Synthesis of Poly-2PACz

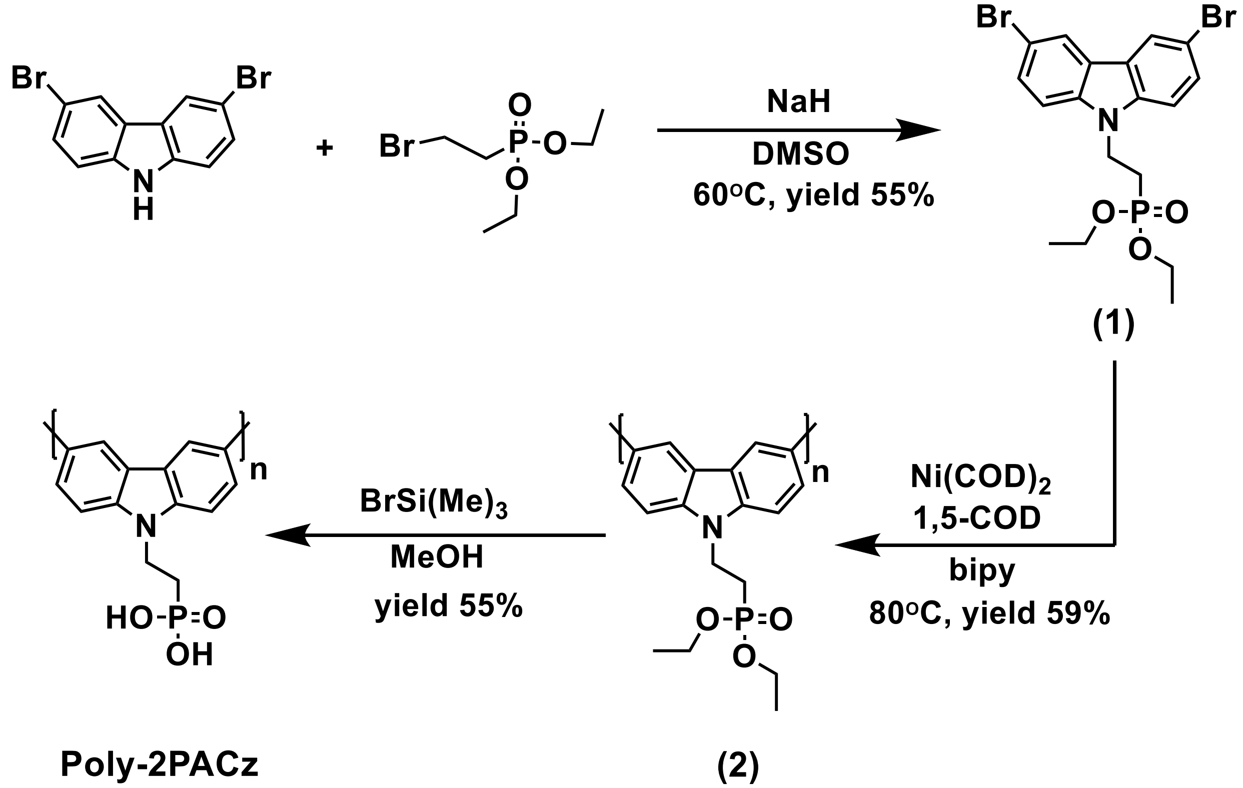

Scheme 1. The synthesis route of Poly-2PACz.

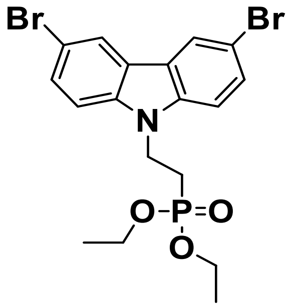

Diethyl(2-(3,6-dibromo-9H-carbazol-9-yl)ethyl)phosphonate (1): 3,6-Dibromo-9H-carbazole (2 g, 6.15 mmol) was dissolved in 20 ml DMSO, then NaH (177 mg, 7.38 mmol) was added slowly and the reaction was stirred at room temperature for 0.5 h. Diethyl (2-bromoethyl)phosphonate (1.2 ml) was added and the reaction was stirred at 60 °C for 24 h. After that, the reaction was cooled to room temperature, and ice water and 1 M HCl were added to adjust pH to 2. The reaction was extracted by EA and washed by DI water. The crude product was purified by column chromatography (DCM and PE:EA=1:1, v/v) to give 1.32 g (55%) of white powder.

^{1}H NMR (400 MHz, $CDCl_3$, ppm): 8.14 (d, J = 2.1 Hz, 2H), 7.54 (dd, J = 8.4 Hz, 2 Hz, 2H), 7.32 (d, J = 8.5 Hz, 2H), 4.57 (m, 2H), 4.04 (m, 4H), 2.2 (m, 2H), 1.24 (t, J = 6.9 Hz, 6H).
^{13}C NMR (400 MHz, $CDCl_3$, ppm):138.7, 129.3, 123.8, 123.4, 112.6, 110.4, 62.0, 37.3, 25.7, 16.4.

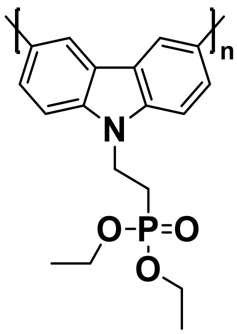

Poly-diethyl(2-(9H-carbazol-9-yl)butyl)phosphonate (2): $Ni(COD)_2$ (0.36 g, 1.30 mmol), 2,2'-bipyridine (0.20 g, 1.30 mmol), and cyclooctadiene (0.16 ml, 1.30 mmol) were stirred in 10 ml DMF at 80 °C for 0.5 hours, and then compound 1 (0.528 g, 1.08 mmol) was added to the reaction mixture. Then, the reaction mixture was stirred overnight at 80 °C, cooled to room temperature and 1M HCl was added to adjust pH to 1-2. The resulting precipitate was isolated by filtration. The polymer was added to dichloromethane and reprecipitated into diethyl ether, and washed successively with 200 ml of hot 0.01 M EDTA solution (pH = 3-4), 200 ml of hot 0.01 M EDTA solution (pH = 8-9), and 200 ml of water. The polymer was then dried under vacuum to give 0.21 g (59%) light yellow powder.
^{1}H NMR (400 MHz, $CDCl_3$, ppm): 7.35-8.89 (m, 6H), 4.35-4.77 (m, 2H), 3.90-4.28 (m, 4H), 2.15-2.43 (m, 2H), 1.12-1.38 (m, 6H).
GPC analysis (THF vs. PS): M_n = 4052 g mol^{-1}, M_w = 5289 g mol^{-1}; PDI = 1.3.

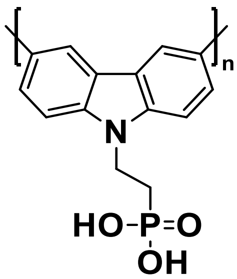

Poly-2PACz: compound 2 (0.05 g) was dissolved in 10 ml CH_2Cl_2 and $BrSi(Me)_3$ (0.07 g) was added to the solution. The reaction mixture was then stirred at room temperature for 12 h and then

5 ml methanol was added and stirred for 12 h. The solution was concentrated and dropped into the ether to precipitate to give 25 mg brown solid (55%). It should be noted that phosphonate groups may not fully convert to phosphonic acid during the hydrolysis process. In addition, Poly-2PACz is not soluble in common GPC solvents like DMF and THF, and direct molecular weight determination via GPC is not feasible. Given that the hydrolysis reaction occurs exclusively at the phosphate side chains, we can reasonably estimate the molecular weight of Poly-2PACz to be approximately 3549 Da based on the polymerization degree of compound 2. The NMR and GPC spectra related to the synthesis of Poly-2PACz are shown in Supplementary Figs. S16 to S19.

Supplementary Figures and Table

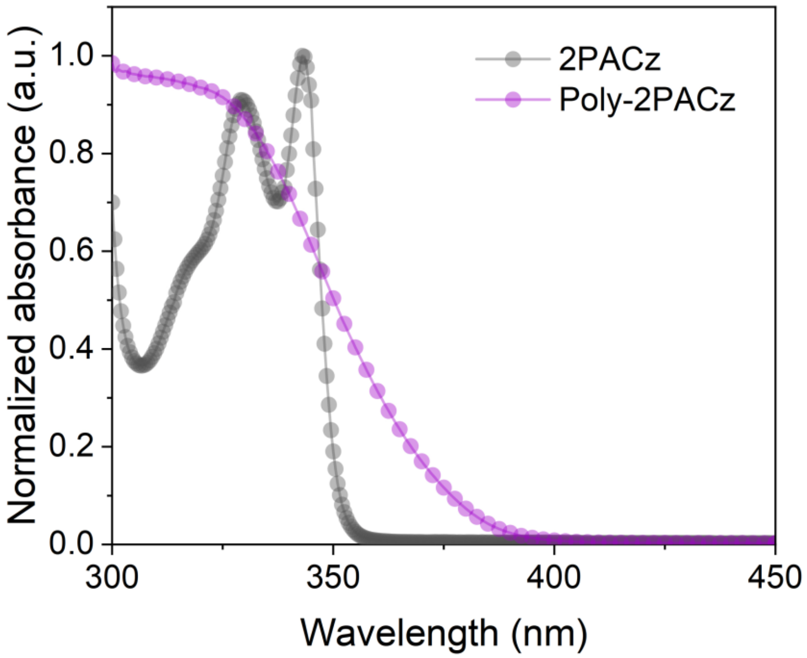

Fig. S1. UV-vis absorption spectra of the 2PACz and Poly-2PACz thin films.

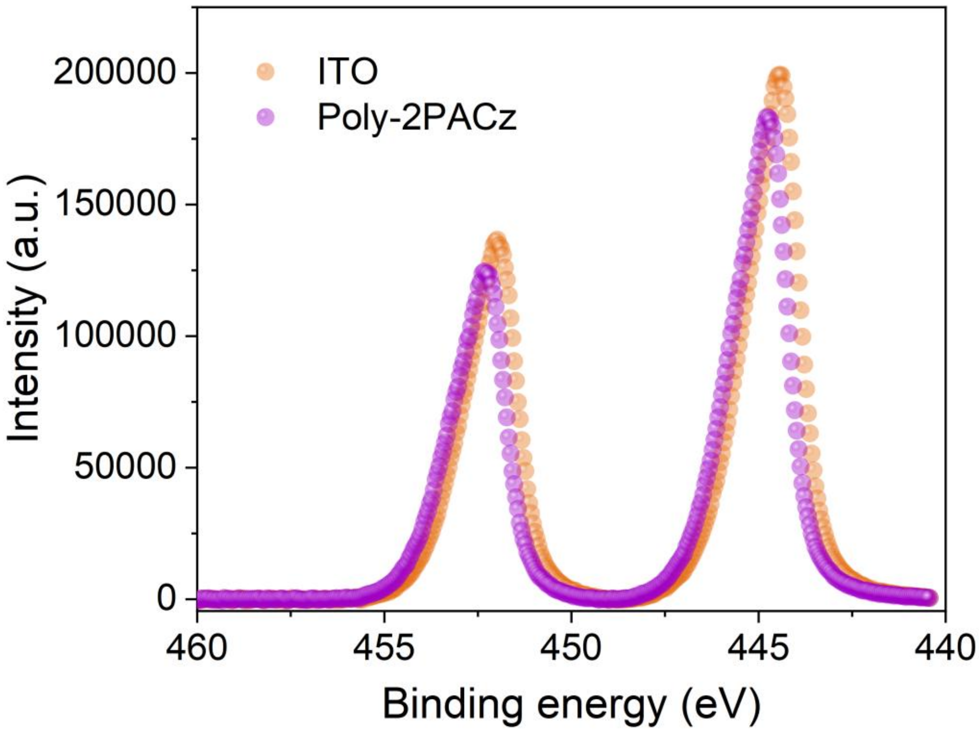

Fig. S2. XPS In 3d spectra of bare ITO and ITO/Poly-2PACz.

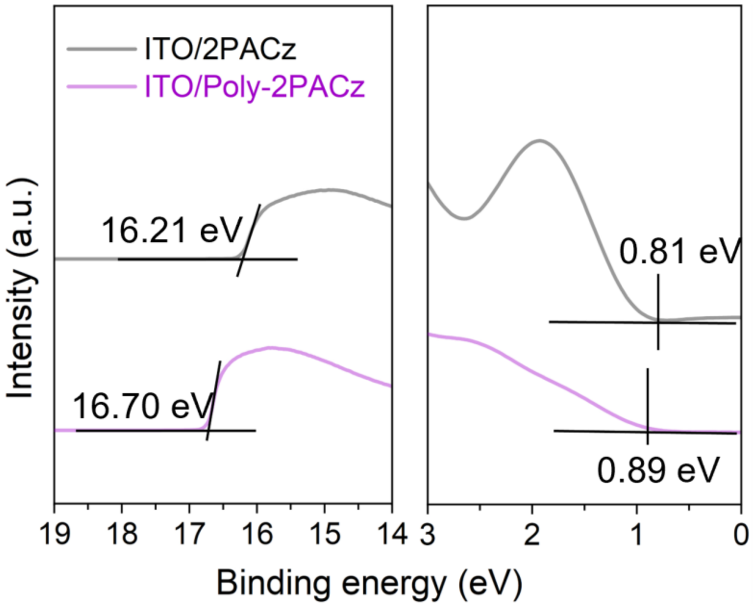

Fig. S3. UPS spectra of 2PACz and Poly-2PACz thin films on ITO glass substrates.

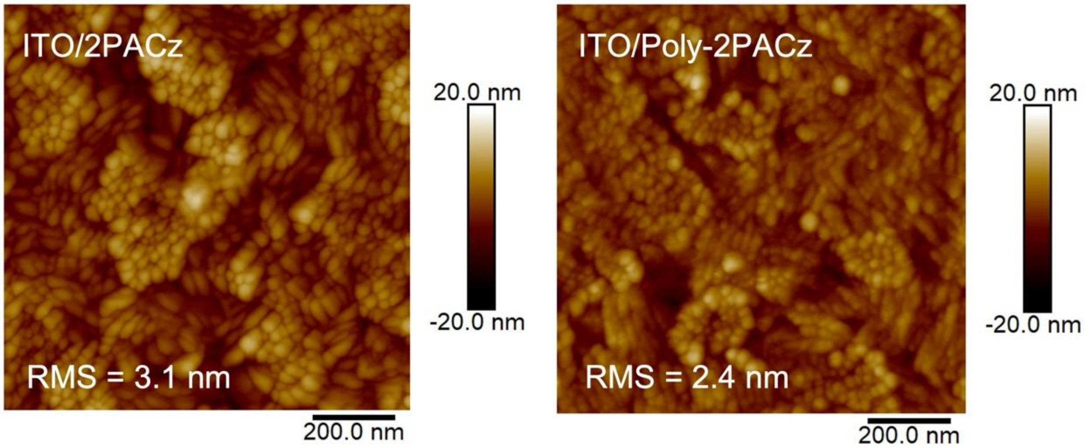

Fig. S4. AFM height images of the ITO glass substrates coated with 2PACz and Poly-2PACz.

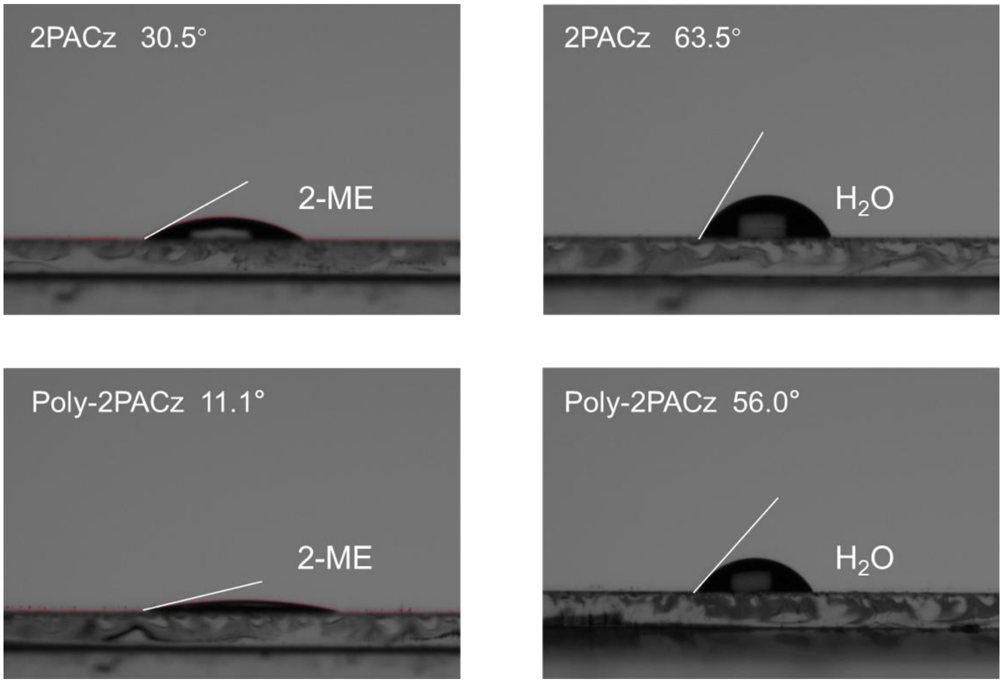

Fig. S5. Contact angle measurements of water and 2-ME on the ITO glass substrates coated with 2PACz and Poly-2PACz.

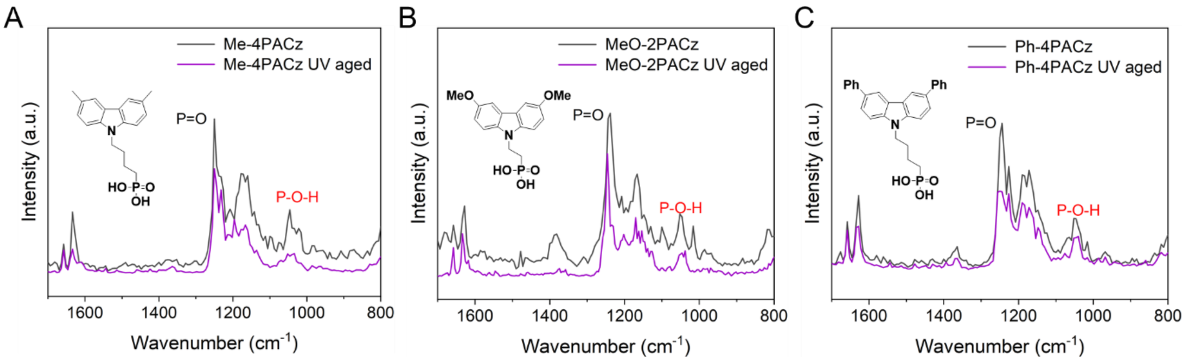

Fig. S6. IR-PiFM spectra of Me-4PACz (A), MeO-2PACz (B), and Ph-4PACz (C) thin films on Si/ITO substrates before and after 24-hour UV irradiation (365 nm, 17.0 mW cm^{-2}).

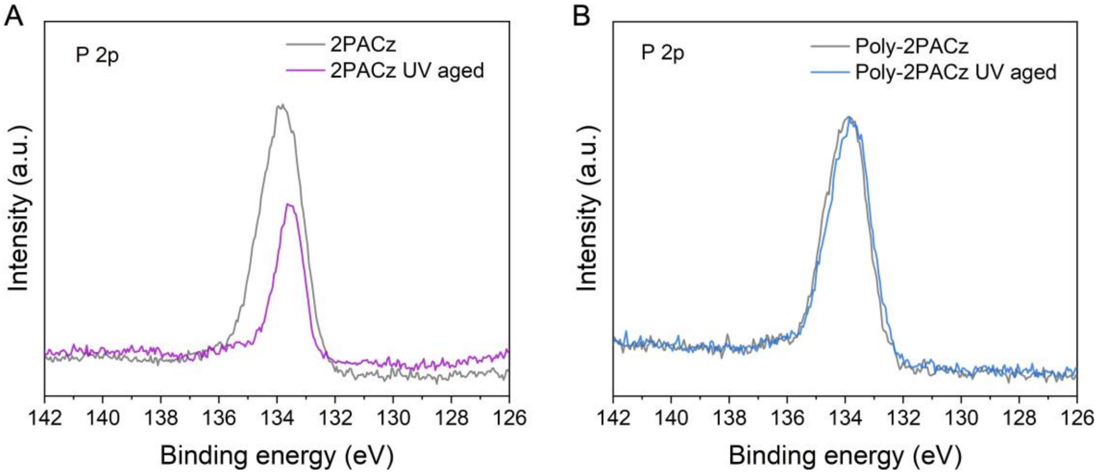

Fig. S7. XPS P 2p spectra of the ITO substrates covered by 2PACz (A) and Poly-2PACz (B) before and after UV irradiation.

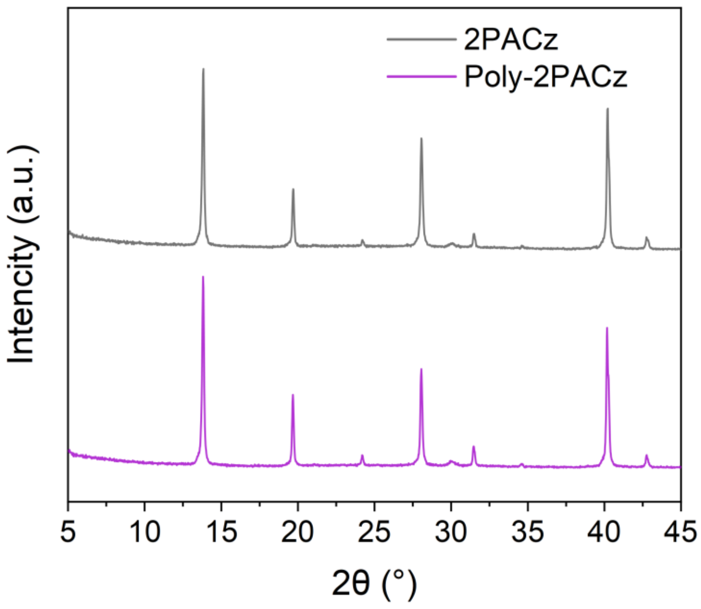

Fig. S8. XRD patterns of the perovskite films coated on 2PACz and Poly-2PACz.

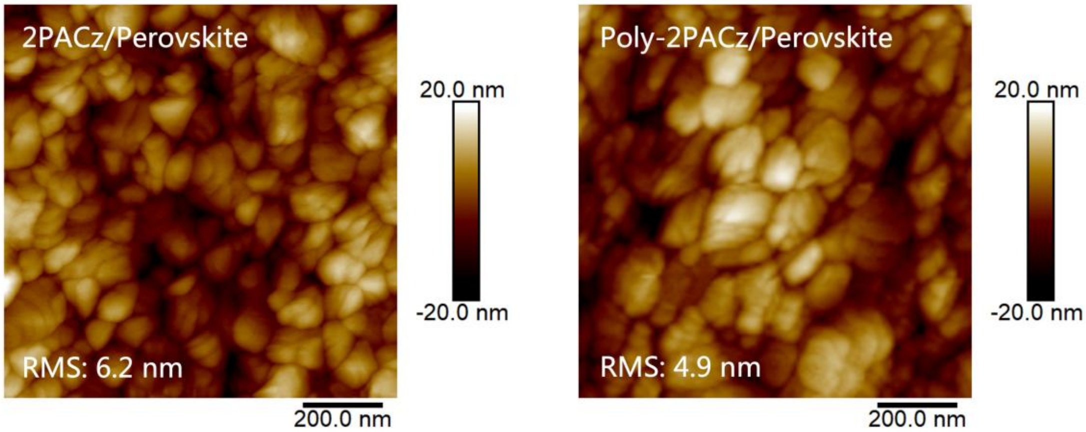

Fig. S9. AFM height images of 2PACz- and Poly-2PACz-based perovskite films.

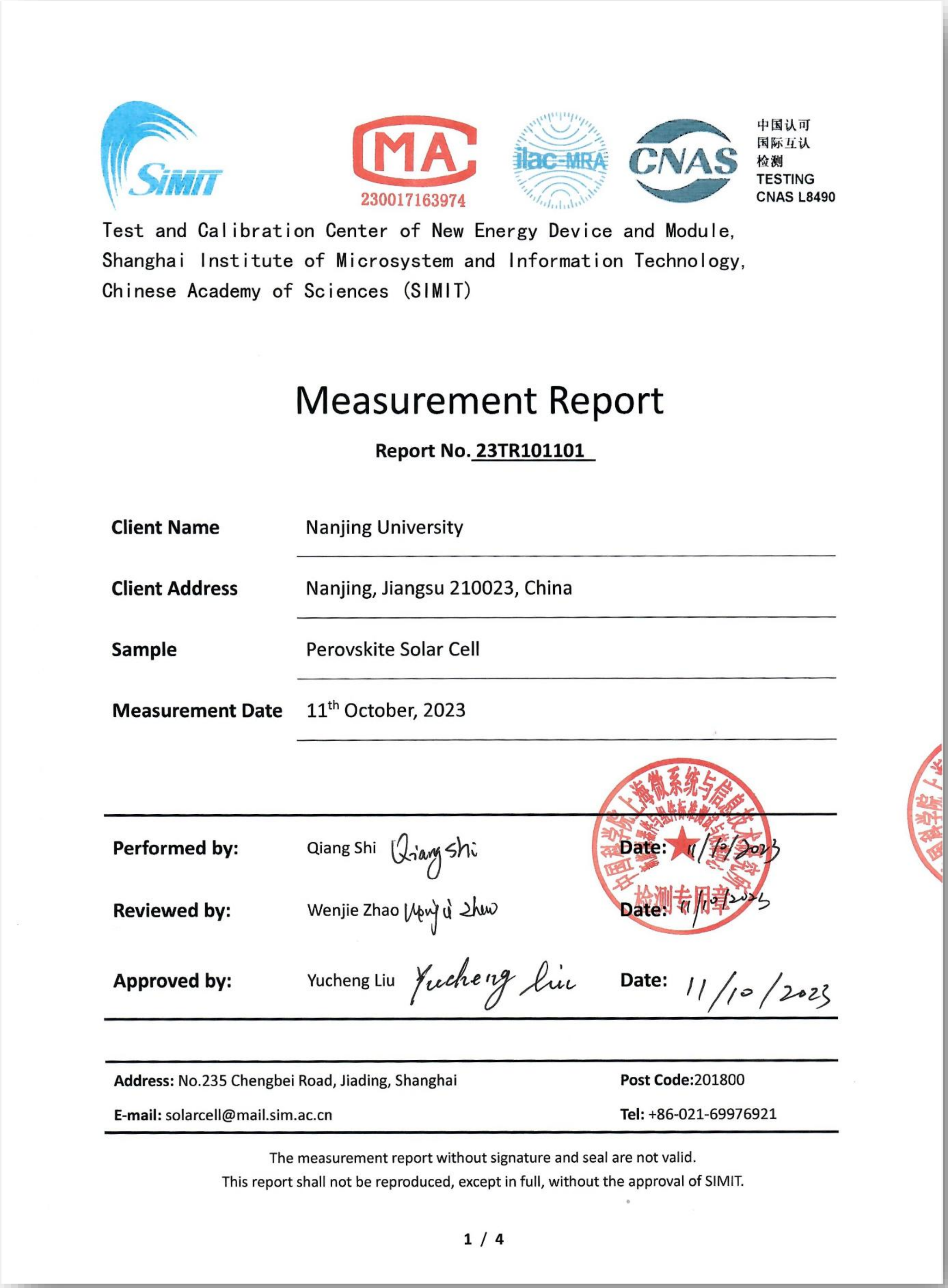

中国认可
国际互认
检测
TESTING
CNAS L8490

230017163974

Test and Calibration Center of New Energy Device and Module,
Shanghai Institute of Microsystem and Information Technology,
Chinese Academy of Sciences (SIMIT)

Measurement Report

Report No. 23TR101101

**Client Name**	Nanjing University
**Client Address**	Nanjing, Jiangsu 210023, China
**Sample**	Perovskite Solar Cell
**Measurement Date**	11th October, 2023

**Performed by:**	Qiang Shi Qiang Shi	**Date:** 11/10/2023
**Reviewed by:**	Wenjie Zhao Wenjie Zhao	**Date:** 11/10/2023
**Approved by:**	Yucheng Liu Yucheng Liu	**Date:** 11/10/2023

Address: No.235 Chengbei Road, Jiading, Shanghai **Post Code:**201800
E-mail: solarcell@mail.sim.ac.cn **Tel:** +86-021-69976921

The measurement report without signature and seal are not valid.
This report shall not be reproduced, except in full, without the approval of SIMIT.

1 / 4

Fig. S10-1. Certification report of Poly-2PACz-based PSC measured at the Shanghai Institute of Microsystem and Information Technology (SIMIT), Chinese Academy of Sciences.

Report No. 23TR101101

Sample Information

Sample Type	Perovskite solar cell
Serial No.	3-2#
Lab Internal No.	23101101-1#
Measurement Item	I-V characteristic
Measurement Environment	24.3±2.0°C,48.6±5.0%R.H

Measurement of I-V characteristic

**Reference cell**	PVM 1121
**Reference cell Type**	mono-Si, WPVS, calibrated by NREL (Certificate No. ISO 2075)
**Calibration Value/Date of Calibration for Reference cell**	144.53mA/ Feb. 2023
**Measurement Conditions**	Standard Test Condition (STC):<br>Spectral Distribution: AM1.5 according to IEC 60904-3 Ed.3,<br>Irradiance: 1000±50W/m^2, Temperature: 25±2°C
**Measurement Equipment/ Date of Calibration**	AAA Steady State Solar Simulator (YSS-T155-2M) / July.2023<br>IV test system (ADCMT 6246) / June. 2023<br>SR Measurement system (CEP-25ML-CAS) / April.2023<br>Measuring Microscope (MF-B2017C) / July.2023
**Measurement Method**	I-V Measurement:<br>Linear sweep in direct direction based on IEC 60904-1:2020;<br>Spectral Mismatch factor was calculated according to IEC 60904-7 and I-V correction according to IEC 60891;
**Measurement Uncertainty**	Area: 1.0%(k=2); Isc: 1.9%(k=2); Voc: 1.0%(k=2);<br>Pmax: 2.4%(k=2); Eff: 2.5%(k=2)

2 / 4

Fig. S10-2. Certification report of Poly-2PACz-based PSC measured at the Shanghai Institute of Microsystem and Information Technology (SIMIT), Chinese Academy of Sciences.

SIMIT

Report No. 23TR101101

====Measurement Results ====

	Forward Scan (Isc to Voc)		Reverse Scan (Voc to Isc)	
Area	7.58 mm^2			
Isc	1.936	mA	1.938	mA
Voc	1.196	V	1.199	V
Pmax	1.896	mW	1.911	mW
Ipm	1.823	mA	1.825	mA
Vpm	1.040	V	1.041	V
FF	81.90	%	82.25	%
Eff	25.02	%	25.21	%

- Spectral Mismatch Factor: SMM=0.9935.
- Designated illumination area defined by a thin mask was measured by measuring microscope.
- Test results listed in this measurement report refer exclusively to the mentioned measured sample.
- The results apply only at the time of the test, and do not imply future performance.

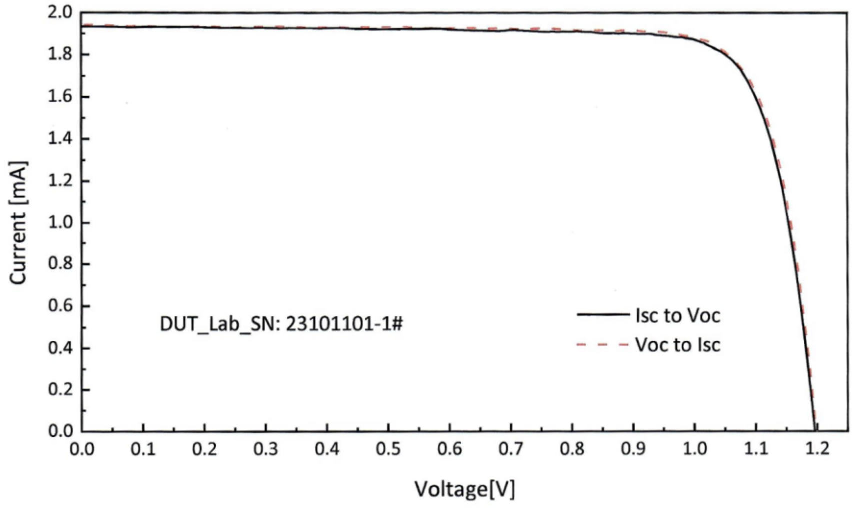

Fig.1 I-V curves of the measured sample

Fig. S10-3. Certification report of Poly-2PACz-based PSC measured at the Shanghai Institute of Microsystem and Information Technology (SIMIT), Chinese Academy of Sciences.

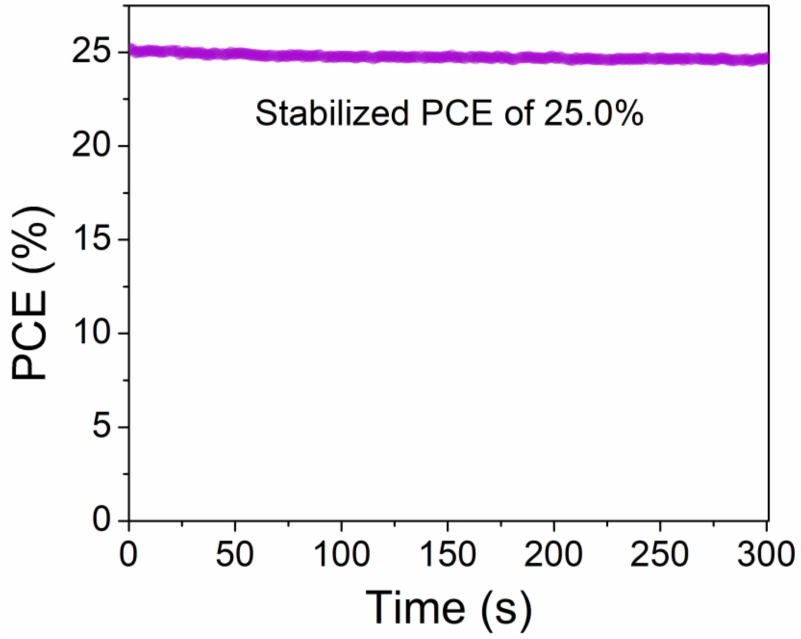

Fig. S10-4. The 300-second MPP tracking curve of the Poly-2PACz-based PSC measured at the Shanghai Institute of Microsystem and Information Technology (SIMIT), Chinese Academy of Sciences.

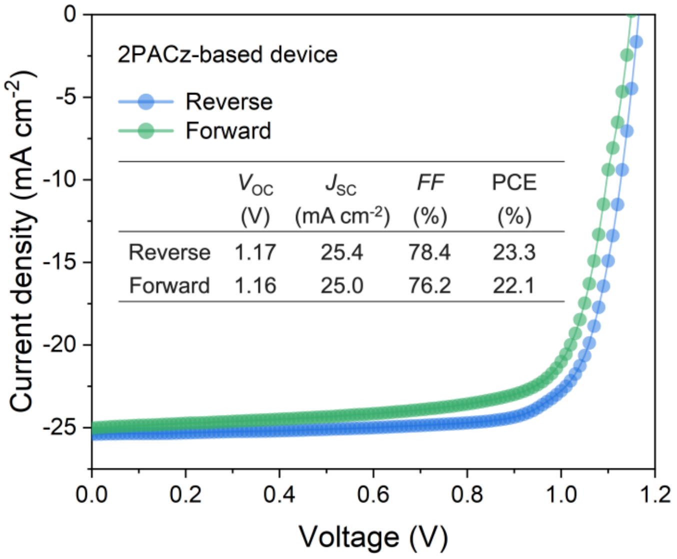

Fig. S11. Reverse and forward *J-V* characteristic curves of the PSCs based on 2PACz. Inset shows the device parameters derived from the curves.

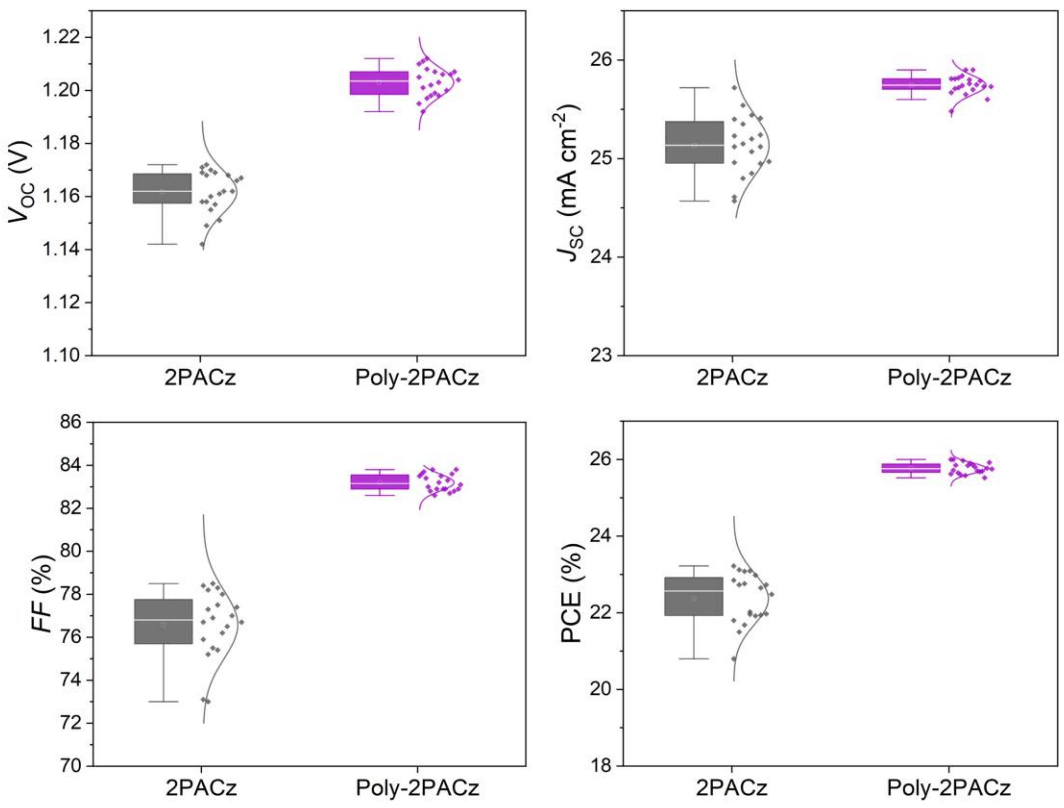

Fig. S12. Statistical results of PCE, V_{OC}, J_{SC}, and *FF* distributions for the devices based on 2PACz and Poly-2PACz.

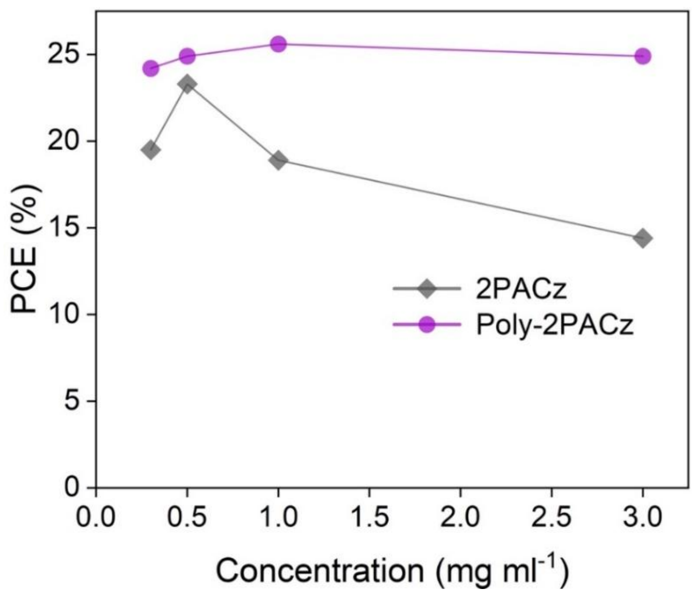

Fig. S13. PCE versus HTL solution concentration curves of 2PACz- and Poly-2PACz-based PSCs.

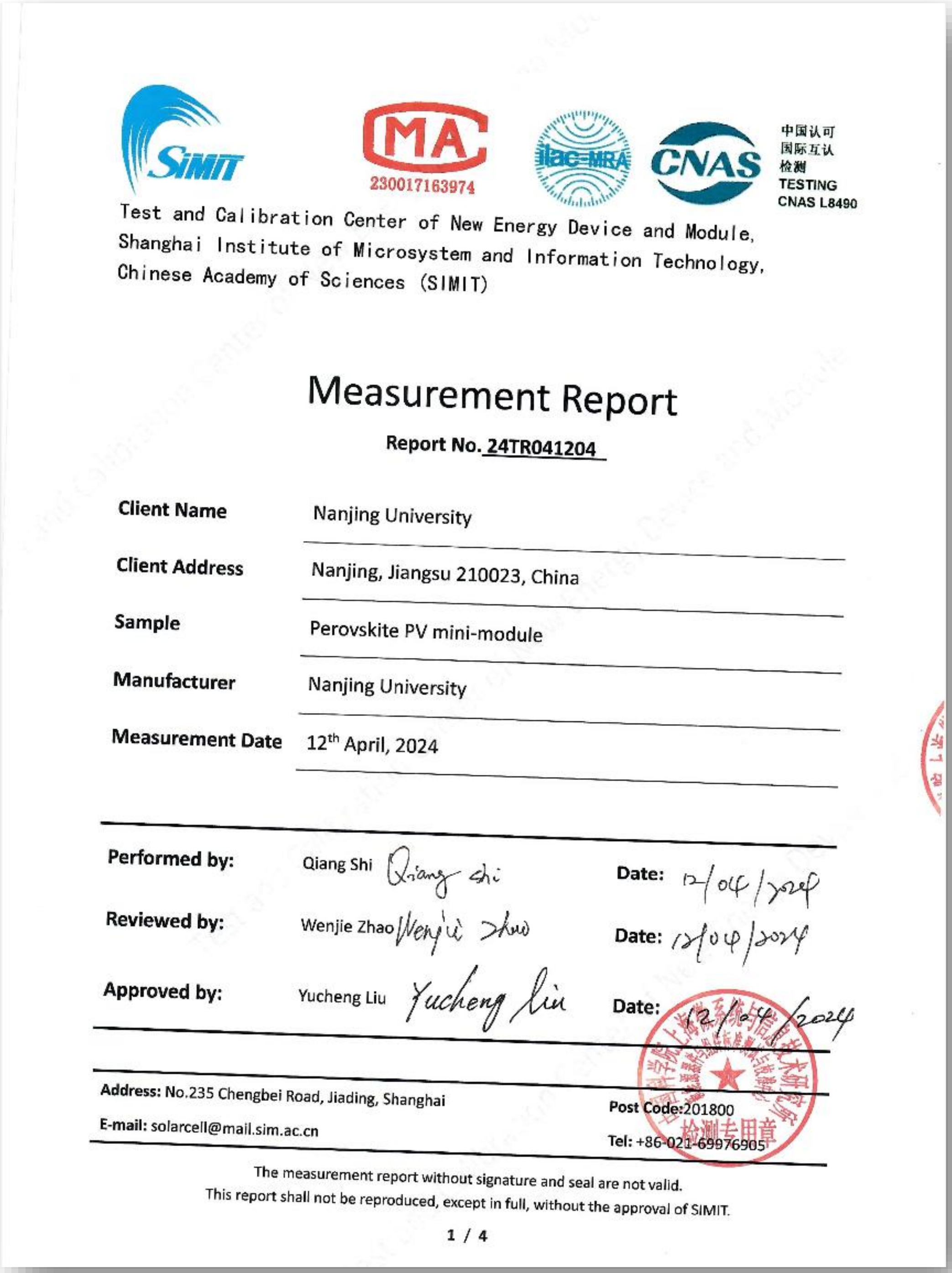
CMA 230017163974

中国认可
国际互认
检测
TESTING
CNAS L8490

Test and Calibration Center of New Energy Device and Module,
Shanghai Institute of Microsystem and Information Technology,
Chinese Academy of Sciences (SIMIT)

Measurement Report

Report No. 24TR041204

**Client Name**	Nanjing University
**Client Address**	Nanjing, Jiangsu 210023, China
**Sample**	Perovskite PV mini-module
**Manufacturer**	Nanjing University
**Measurement Date**	12th April, 2024

**Performed by:**	Qiang Shi Qiang Shi	**Date:**	12/04/2024
**Reviewed by:**	Wenjie Zhao Wenjie Zhao	**Date:**	12/04/2024
**Approved by:**	Yucheng Liu Yucheng Liu	**Date:**	12/04/2024

Address: No.235 Chengbei Road, Jiading, Shanghai
Post Code:201800
E-mail: solarcell@mail.sim.ac.cn
Tel: +86-021-69976905

检测专用章

The measurement report without signature and seal are not valid.
This report shall not be reproduced, except in full, without the approval of SIMIT.

1 / 4

Fig. S14-1. Certification report of Poly-2PACz-based minimodule measured at the Shanghai Institute of Microsystem and Information Technology, Chinese Academy of Sciences (SIMIT).

SIMIT

Report No. 24TR041204

Sample Information	
Sample Type	Perovskite PV mini-module
Serial No.	3-1
Lab Internal No.	24041202-3#
Measurement Item	I-V characteristic
Measurement Environment	24.7±2.0°C, 36.6±5.0%R.H

Measurement of I-V characteristic	
**Reference cell**	PVM1121
**Reference cell Type**	mono-Si, WPVS, calibrated by NREL (Certificate No. ISO 2098)
**Calibration Value/Date of Calibration for Reference cell**	143.95mA/ Feb. 2024
**Measurement Conditions**	Standard Test Condition (STC):<br>Spectral Distribution: AM1.5 according to IEC 60904-3 Ed.3,<br>Irradiance: 1000±50W/m², Temperature: 25±2°C
**Measurement Equipment/ Date of Calibration**	AAA Steady State Solar Simulator (YSS-T155-2M) / July.2023<br>IV test system (ADCMT 6246) / June. 2023<br>Measuring Microscope (MF-B2017C) / July.2023<br>SR Measurement system (CEP-25ML-CAS) / April.2023
**Measurement Method**	I-V Measurement:<br>Logarithmic sweep in both directions (Voc to Isc and Isc to Voc) during one flash based on IEC 60904-1:2020;<br>Spectral Mismatch factor was calculated according to IEC 60904-7 and I-V correction according to IEC 60891.
**Measurement Uncertainty**	Area: 1.0%(k=2); Isc: 2.0%(k=2); Voc: 1.0%(k=2);<br>Pmax: 2.5%(k=2); Eff: 2.6%(k=2)

2 / 4

Fig. S14-2. Certification report of Poly-2PACz-based minimodule measured at the Shanghai Institute of Microsystem and Information Technology, Chinese Academy of Sciences (SIMIT).

Report No. 24TR041204

====Measurement Results ====

	Forward Scan (Isc to Voc)		Reverse Scan (Voc to Isc)	
Area	1272.33 mm²			
Isc	48.485	mA	48.485	mA
Voc	7.261	V	7.247	V
Pmax	268.430	mW	274.919	mW
Ipm	43.494	mA	43.417	mA
Vpm	6.172	V	6.347	V
FF	76.28	%	78.24	%
Eff	21.10	%	21.61	%

- Spectral Mismatch Factor SMM_{top}=0.9980.
- Designated Illumination area defined by the black tape was measured by a measuring.
- Test results listed in this measurement report refer exclusively to the mentioned test sample.
- The results apply only at the time of the test, and do not imply future performance.

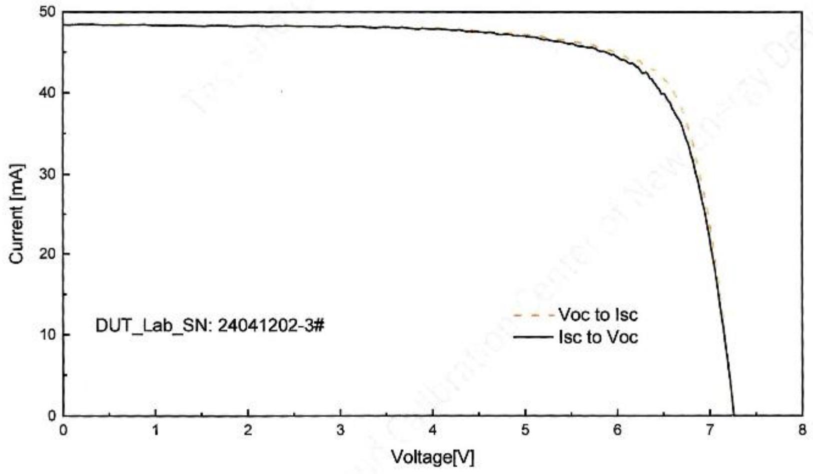

Fig.1 I-V curves of the measured sample

Fig. S14-3. Certification report of Poly-2PACz-based minimodule measured at the Shanghai Institute of Microsystem and Information Technology, Chinese Academy of Sciences (SIMIT).

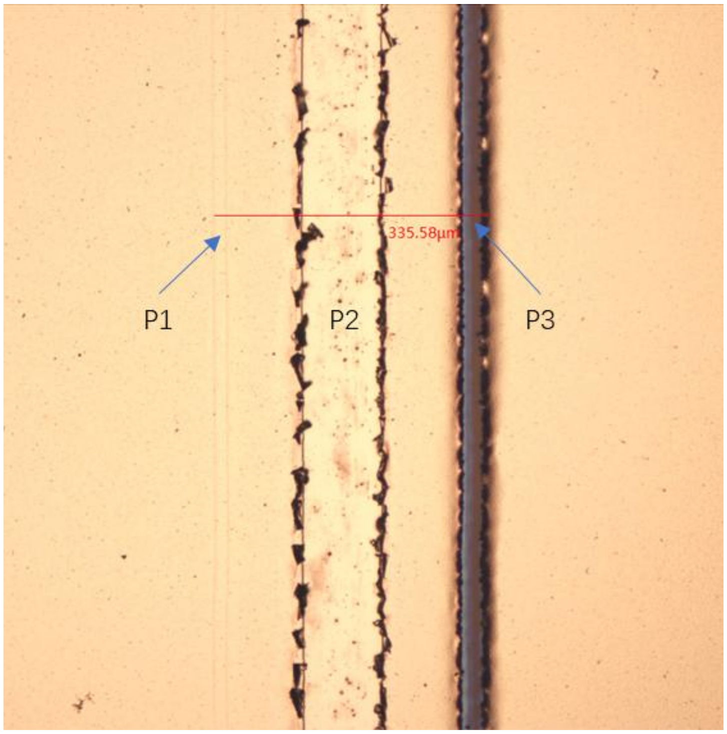

Fig. S15. Optical image of the module dead area obtained with a metallographic microscope.

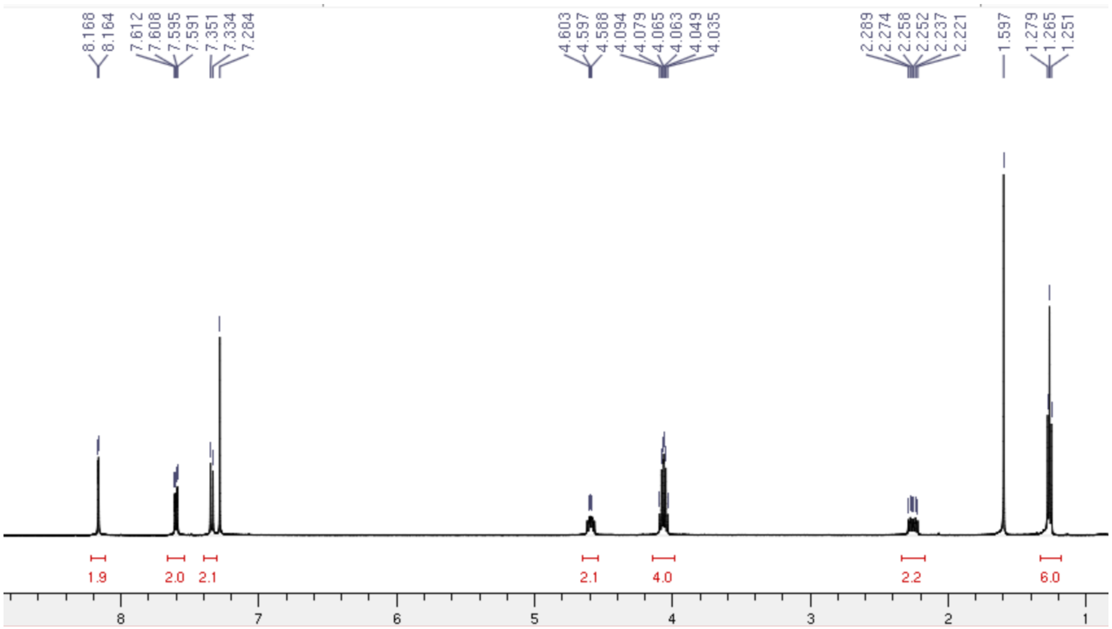

Fig. S16. ^{1}H NMR spectrum of compound 1 in $CDCl_3$.

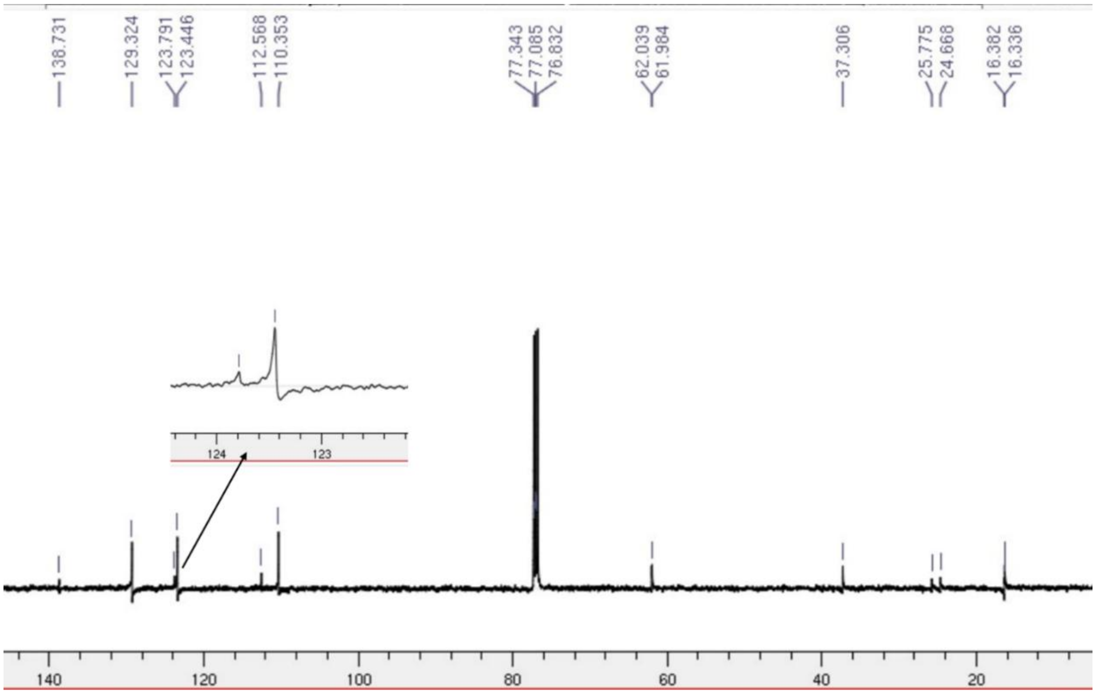

Fig. S17. ^{13}C NMR spectrum of compound 1 in $CDCl_3$.

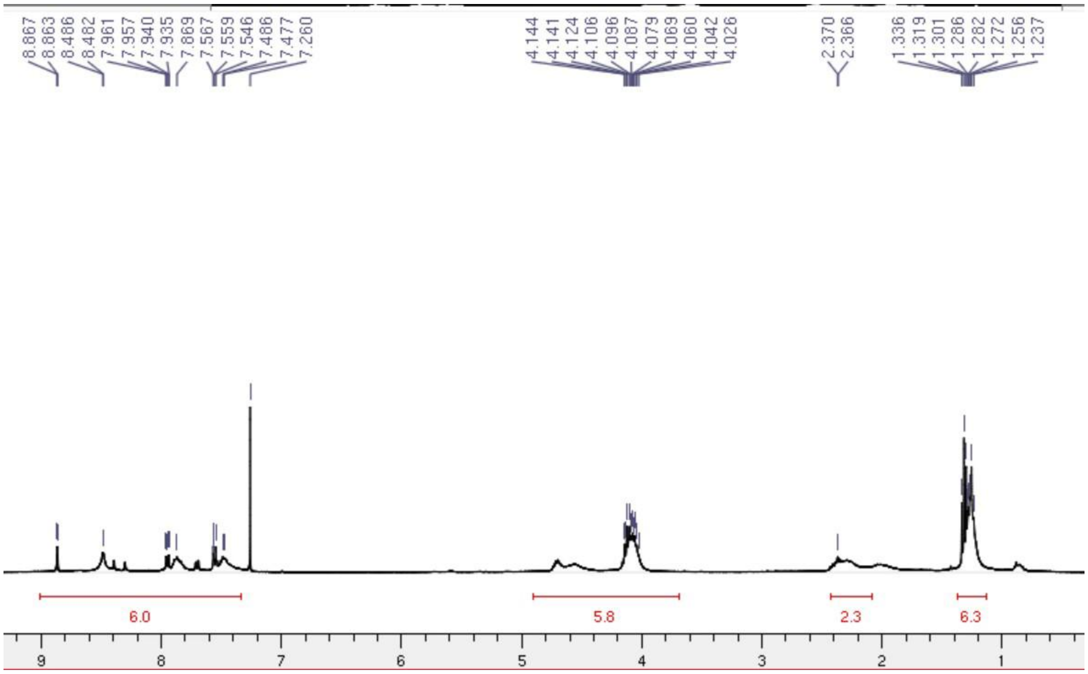

Fig. S18. ^{1}H NMR spectrum of compound 2 in $CDCl_3$.

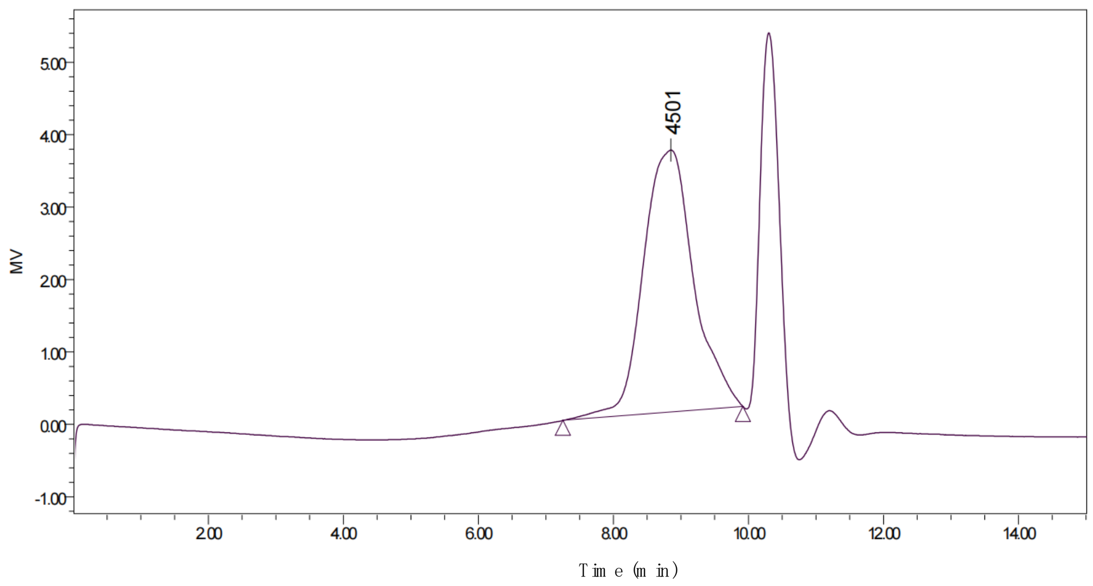

Fig. S19. GPC spectrum of compound 2.

Table S1. Summary of the fitting parameters for the TRPL spectra using the bi-exponential decay function $y = y_0 + A_1 exp(-x/t_1) + A_2 exp(-x/t_2)$, $t_{average} = (A_1 t_1^2 + A_2 t_2^2)/(A_1 t_1 + A_2 t_2)$.

Sample	A_1	t_1 (ns)	A_2	t_2 (ns)	$t_{average}$ (ns)
2PACz/Perovskite	0.383	28.6	0.209	1602	1552
Poly-2PACz/Perovskite	0.394	23.3	0.244	2944	2907

Table S2. Statistical analysis of PSC photovoltaic parameters based on 2PACz and Poly-2PACz.

HTL	V_{OC} (V)	J_{SC} (mA cm^{-2})	FF (%)	PCE (%)
2PACz	1.17<br>(1.16±0.01)	25.4<br>(25.1±0.3)	78.4<br>(76.8±1.6)	23.3<br>(22.6±0.7)
Poly-2PACz	1.21<br>(1.20±0.01)	25.7<br>(25.7±0.1)	83.7<br>(83.2±0.4)	26.0<br>(25.9±0.1)
